# β-Cryptoxanthin Confers Radioprotection against Intestinal Injury via NRF2-Mediated Antioxidant Response and Gut Microbiota Reprogramming

**DOI:** 10.7150/ijbs.125307

**Published:** 2026-02-11

**Authors:** Manman Zhang, Yingshuang Liu, Wenbo Ma, Jinhan Wang, Ningning He, Huijuan Song, Yeqing Gu, Mengmeng Yang, Xinran Lu, Jingxing Sun, Chang Xu, Liqing Du, Yayi Yuan, Yan Wang, Kaihua Ji, Qiang Liu

**Affiliations:** 1 Institute of Radiation Medicine, Chinese Academy of Medical Sciences & Peking Union Medical College, Tianjin Key Laboratory of Radiation Medicine and Molecular Nuclear Medicine, Tianjin Institutes of Health Science, State Key Laboratory of Advanced Medical Materials and Devices, Tianjin 300192, China.; 2 Wenzhou Institute University of Chinese Academy of Sciences, Wenzhou 325000, China.; 3 Department of Anatomy, Shandong Second Medical University, Weifang 261053, China.; 4 CNNC Key Laboratory on Radio-Toxicology and Radiopharmaceutical Preclinical Evaluation, Shanxi Key Laboratory of Drug Toxicology and Preclinical Studies for Radiopharmaceutical, Taiyuan 030006, China.; 5 School of Population Medicine and Public Health, Chinese Academy of Medical Sciences & Peking Union Medical College, Beijing 100730, China.

**Keywords:** β-Cryptoxanthin, Radiation-induced intestinal injury, NRF2 signaling, Radioprotection, Gut microbiota

## Abstract

Radiation-induced intestinal injury, a common complication of abdominal/pelvic radiotherapy for cancer patients and accidental irradiation, presents a major clinical challenge due to the lack of effective treatments. This study investigates the radioprotective potential of β-cryptoxanthin, a provitamin A carotenoid known for its antioxidant properties. *In vivo*, oral β-cryptoxanthin administration alleviated radiation-induced intestinal injury by enhancing the NRF2-mediated antioxidant response, which was confirmed by its lack of efficacy in *Nrf2^-/-^* mice. Additionally, it restored radiation-impaired microbiota by increasing beneficial bacterial populations and protective metabolites like short-chain fatty acids (SCFAs), thereby re-establishing a radioprotective gut ecosystem. At the cellular level, β-cryptoxanthin pretreatment significantly improved cell viability and proliferation while reducing reactive oxygen species (ROS), apoptosis, and DNA damage in irradiated MODE-K intestinal epithelial cells. Mechanistically, β-cryptoxanthin activated the AMPK-GSK3β signaling axis, which drove NRF2 nuclear translocation and upregulated NRF2-dependent cytoprotective genes. Knockdown of NRF2 or AMPK abolished the radioprotective effects, confirming the involvement of these pathways. Overall, this study demonstrates that β-cryptoxanthin protects against radiation-induced intestinal injury through dual mechanisms: activating the NRF2-mediated antioxidant response and reprogramming the gut microbiota to restore a radioprotective ecosystem. These findings position β-cryptoxanthin as a promising candidate for clinical radioprotection.

## Introduction

Radiation therapy remains a cornerstone in the management of abdominal and pelvic malignancies, including colorectal, cervical, and prostate cancers, and is integral to both curative and palliative treatment strategies 1. While it offers substantial benefits in local tumor control and survival, its therapeutic potential is often constrained by collateral injury to radiosensitive normal tissues. Among these, the gastrointestinal tract—particularly the intestines—exhibits marked vulnerability to ionizing radiation (IR). Clinically, 60-80% of patients undergoing abdominal or pelvic radiotherapy develop radiation-induced intestinal injury (RIII), characterized by mucosal inflammation, crypt cell apoptosis, microvascular damage, and compromised barrier integrity 2, 3. Despite its prevalence and clinical impact, there are currently no standardized interventions capable of preventing or effectively reversing RIII 4, 5, underscoring an urgent need for the development of novel and effective radioprotective strategies.

The pathogenesis of RIII involves complex interactions between radiation-induced oxidative stress, cell damage, and inflammatory responses 6. Specifically, radiation-generated reactive oxygen species (ROS) induce DNA damage, protein oxidation, and lipid peroxidation, which trigger cell death and exacerbate inflammatory responses, ultimately worsening tissue injury 7, 8. Additionally, the gut microbiota plays a crucial role in modulating the severity of radiation-induced damage 9. Dysbiosis, or the imbalance of gut microbial communities, is commonly observed following radiation exposure and further amplifies intestinal injury through impaired barrier function and sustained inflammation 10, 11. Targeted microbiota modulation (e.g., via bioactive compounds) can enhance beneficial taxa abundance and protective metabolite production, conferring radioprotection 12. Thus, dual-targeting strategies that simultaneously scavenge ROS and reprogram microbiota may offer synergistic protection against RIII.

Carotenoids are essential dietary nutrients that impart yellow, orange, or red pigmentation of various foods and function as natural antioxidants 13, 14. β-Cryptoxanthin, a provitamin A carotenoid with health benefits, is abundant in citrus fruits and also found in corn, peas, and animal-derived foods such as egg yolk 15, 16. It is one of the six major carotenoids in human plasma 17, 18. β-Cryptoxanthin directly scavenges various types of ROS and reactive nitrogen species, thereby mitigating oxidative stress and protecting lipids, proteins, and nucleic acids against ROS-induced damages 19-21. In cigarette smoke-exposed ferrets, it reduced lung inflammation and oxidative stress, confirming its anti-inflammatory and antioxidant roles 22. Similarly, in *Caenorhabditis elegans*, it exerted an antioxidant activity on nematodes and enhanced antioxidant response 23. Moreover, the beneficial effects of β-cryptoxanthin are also evident in dietary studies. Epidemiologic studies demonstrate that dietary β-cryptoxanthin confers protection against inflammation by reducing oxidative damage via its antioxidant potential, and modestly increased intake is associated with reduced risk of inflammatory disorders such as rheumatoid arthritis 24. Additionally, emerging evidence indicates carotenoids are promising in the modulation of gut microbiota by enriching beneficial bacteria, particularly those producing short chain fatty acids (SCFAs), thereby protecting villi structure and strengthening intestinal barrier integrity 25, 26. Collectively, these ROS-scavenging, anti-inflammatory, and microbiota-modulating properties position β-cryptoxanthin as a promising candidate for mitigating radiation-induced intestinal injury.

While β-cryptoxanthin's antioxidant effects are well-established in cardiovascular, ocular disorders, and oxidative stress-induced skin damage 21, 27, 28, its role in protecting against RIII remains underexplored. In this study, we investigated the radioprotective effects of β-cryptoxanthin and the underlying mechanisms *in vivo* and *in vitro*. We show that β-cryptoxanthin mitigated RIII by activating the NRF2-antioxidant axis and reprogramming the gut microbiota in mice. *In vitro*, β-cryptoxanthin protects MODE-K cells from irradiation by promoting NRF2 nuclear translocation through the activation of the AMPK-GSK3β signaling pathway. These findings highlight β-cryptoxanthin as a promising candidate for radioprotection in abdominal and pelvic radiotherapy.

## Materials and Methods

### Cell lines and cell culture conditions

Mouse intestinal epithelial cell line MODE-K (BNCC338300) was purchased from the BeNa Culture Collection (Beijing, China). Human intestinal epithelial cell line HIEC-6 (CRL-3266), human lung fibroblast cell line MRC-5 (CCL-171), and human renal proximal tubular epithelial cell line HK-2 (CRL-2190) were purchased from the American Type Culture Collection (ATCC, USA). MODE-K and HK-2 cells were cultured in Roswell Park Memorial Institute (RPMI)-1640 medium supplemented with 10% fetal bovine serum (FBS). HIEC-6 and MRC-5 cells were cultured in Dulbecco's Modified Eagle's Medium (DMEM) supplemented with 10% FBS. Cells were incubated at 37 °C in a humidified atmosphere with 5% CO_2_.

### Treatment with β-cryptoxanthin and irradiation

For cell experiment, β-cryptoxanthin (Extrasynthese, France, 0317S) was dissolved in dimethyl sulfoxide (DMSO) and diluted to the required concentration with the corresponding medium. For cytotoxicity determination, MODE-K and HIEC-6 cells were incubated with medium containing different concentrations of β-cryptoxanthin (0.3125, 0.625, 1.25, 2.5, 5, 10, 20, and 40 μM) for 48 h. To evaluate the antioxidation and radioprotective effect of β-cryptoxanthin, MODE-K and HIEC-6 cells were pretreated with 5 μM β-cryptoxanthin for 12 h before exposure to hydrogen peroxide of 400 μM or γ-ray irradiation of 4 Gy or 8 Gy, respectively. A ^137^Cesium γ-ray irradiator at a dose rate of 0.85 Gy/min was used as the irradiation source (Atomic Energy of Canadian Inc., Canada). Control groups received the same amount of DMSO without β-cryptoxanthin.

### Cell viability assay

CCK-8 assay was performed to detect cell viability. For toxicity test, MODE-K or HIEC-6 cells (6 × 10^3^ cells/well) were seeded into 96-well plates and treated with the indicated dose of β-cryptoxanthin for 48 h. For cytoprotective determination, MODE-K or HIEC-6 cells (4 × 10^3^ cells/well) were seeded into 96-well plates and pretreated with 5 μM β-cryptoxanthin or the same volume of DMSO for 12 h before exposure to 8 Gy γ-rays. For SCFA treatment, MODE-K cells (4 × 10^3^ cells/well) were seeded into 96-well plates and pretreated with 5 mM sodium acetate (Sigma-Aldrich, S2889), 1 mM sodium propionate (Sigma-Aldrich, P1880), or 1 mM sodium butyrate (Sigma-Aldrich, 303410) for 24 h before exposure to 8 Gy γ-irradiation. Then, cells were cultured for 24 h and the medium of each well was replaced with 100 μL fresh medium containing 10 μL CCK-8 solution (Meilunbio, China) simultaneously. After incubation at 37 °C for 0.5-2 h in the dark, the absorbance at 450 nm was measured using a multifunctional microplate reader (BioTek Instruments, USA).

### 5-Ethynyl-20-deoxyuridine (EdU) assay

For the EdU assay, MODE-K cells were harvested and analyzed with a EdU Cell Proliferation Kit (RiboBio, China) following the manufacturer's protocol. After incubation with 50 mM EdU for 2 h, cells were washed with PBS and fixed in 4% paraformaldehyde for 30 min. Cells were treated with glycine (2 mg/mL) to neutralize excess aldehyde groups, incubated with 0.5% Triton X-100, and stained with Apollo Dye Solution. The nuclei were stained with 4′,6-diamidino-2-phenylindole (DAPI), and EdU-positive cells were photographed using an EVOS inverted fluorescence microscope (Thermo Fisher, USA).

### ROS detection

Cellular ROS was detected using the total ROS Assay Kit (Thermo Fisher Scientific, USA). MODE-K cells were incubated with ROS probe for 1 h at 37 °C in the dark. Then, cells were washed with PBS to remove the free ROS probe, collected by trypsinization and centrifugation, and resuspended in PBS. ROS levels were measured by fluorescence in the FITC channel using a flow cytometer (Mindray, China).

### Cell apoptosis detection

MODE-K cells were cultured in 6-well plates and pretreated with 5 μM β-cryptoxanthin for 12 h prior to irradiation of 8 Gy. After irradiation, cells were cultured for 48 h and then harvested, washed with PBS and stained using Annexin V-FITC Apoptosis Detection Kit (BD, USA). Cells were stained in the dark for 15 min with annexin V-FITC and propidium iodide, and the percentage of cell apoptosis was analyzed by flow cytometry (Mindray, China).

### Cell cycle analysis

MODE-K cells were pretreated with 5 μM β-cryptoxanthin for 12 h and then exposed to 8 Gy irradiation. At 12 h and 24 h post-irradiation, cells were harvested, washed twice with PBS, and fixed overnight in 75% ice-cold ethanol. After fixation, cells were washed and incubated in a staining solution containing 10 μg/mL RNase A, 50 μg/mL propidium iodide, and 4 mM sodium citrate at 37 °C for 15 min in the dark. Cell cycle distribution was analyzed using a flow cytometer (Mindray, China) and quantified with FlowJo software.

### Immunofluorescence assays

MODE-K cells were seeded onto glass coverslips in 12-well plates and pretreated with 5 μM β-cryptoxanthin for 12 h before exposure to 4 Gy irradiation. Cells were collected at the specified time points, fixed in 4% paraformaldehyde for 15 min and permeabilized with 0.3% Triton X-100 at room temperature for 15 min. After washing three times with cold PBS, samples were blocked with 1% bovine serum albumin (BSA) for 2 h and then incubated with primary antibodies at 4 °C overnight and corresponding fluorescein-conjugated secondary antibodies for 1 h. The nuclei were stained with DAPI. For γH2AX detection, phospho-histone H2AX (Ser139) antibody (Millipore, #05-636) was used as the primary antibody. Images were acquired using an EVOS inverted fluorescence microscope (Thermo Fisher, USA), and the number of γH2AX foci in randomly selected cells from each group were quantified. For NRF2 detection, anti-NRF2 antibody (Proteintech, 16396-1-AP) was used as the primary antibody, and images were acquired using a confocal laser scanning microscope (Leica, Germany). All experiments were repeated in triplicate.

### Quantitative real-time PCR (qPCR) analysis

The total RNA in each group was isolated from MODE-K cells or tissues using TRIzol reagent (Invitrogen, USA) according to the manufacturer's instructions, and equal amounts of RNA were reverse transcribed into cDNA using HiScript^®^ II Q RT SuperMix (Vazyme, China). *Nrf2*, *Ho-1*, *Nqo1*, *Sod1*, *Sod2*, *Cat*, *Gst* and *Gapdh* mRNA transcription levels were determined using TransStart Top Green qPCR SuperMix (Transgen, China) with the Opticon® System (Bio-Rad, USA). The sequences of primers used in this study are listed in **Table S1**. The results were normalized to the *Gapdh* rRNA gene level. The fold change of gene expression was calculated using 2^-∆∆*C*^_T_ method.

### Western blotting

Cells or homogenized tissues were lysed on ice in radioimmunoprecipitation assay (RIPA) lysis buffer (Solarbio, China) mixed with protease and phosphatase inhibitors cocktail (Roche, USA). Equal amounts of total protein extracts were separated by SDS-PAGE and transferred to nitrocellulose membranes (Millipore, USA). The membranes were incubated in blocking buffer for 2 h, probed overnight with primary antibodies, and then incubated with secondary antibodies for 2 h. The protein bands were detected and quantified using the ChemiDoc MP imaging system (Bio-Rad) and Image Lab software (version 6.1, Bio-rad). To determine the nuclear levels of NRF2, the proteins in the cytoplasm and nucleus were isolated using NE-PER Nuclear and Cytoplasmic extraction reagent (Thermo Scientific, USA) and then analyzed by Western blotting, using GAPDH as the cytoplasmic control and Lamin B1 as the nuclear control.

The following primary antibodies were used: anti-HO-1 (abcam, ab13248), anti-NQO1 (abcam, ab80588), anti-AMPK (abcam, ab32047), anti-p-AMPK (abcam, ab133448), anti-NRF2 (Proteintech, 16396-1-AP), anti-Keap1 (Proteintech, 10503-2-AP), anti-GSK3β (CST, #9832), anti-p-GSK3β (Ser9) (CST, #5558), anti-GAPDH (Proteintech, 60004-1-Ig), anti-Lamin B1 (Proteintech, 66095-1-Ig), anti-Tubulin (Proteintech, 66240-1-Ig).

### RNA interference assay

Small interfering RNA (siRNA) that target NRF2 and AMPK, and negative control siRNA were synthesized by GenePharma Co., Ltd. (Shanghai, China) and transfected with lipofectamine RNAiMAX reagent (Invitrogen, USA) according to the kit protocol. NRF2-targeting siRNA sense sequence was 5'-CCGGCAUUUCACUAAACACAA-3'. AMPK-targeting siRNA sense sequence was 5'-GGGAACACGAGUGGUUUAATT-3'. Cells were incubated for 48 h to be transfected, and then collected for cell viability assay, EdU assay, immunofluorescence assays, and western blot assays, respectively.

### Animal experiments

All animal experiments involved in this study have been conducted in accordance with the principles of the Animal Welfare Law, the Implementing Regulations of Animal Welfare, and the Guidelines for the Care and Use of Laboratory Animnals and approved by the Institutional Animal Care and Use Committee of the Institute of Radiation Medicine (IRM) of the Chinese Academy of Medical Sciences (approval number: IRM-DWLL-2023083). Male C57BL/6 mice, 6-8 weeks old (weighing 18-20 g), were purchased from Beijing HFK Bioscience Co., Ltd. (Beijing, China). All mice were maintained in a certified specific pathogen-free (SPF) facility. After 1 week of acclimation, mice were randomly distributed into four groups as follows: (1) control; (2) β-cryptoxanthin; (3) IR; and (4) IR + β-cryptoxanthin, with six mice per group. In the β-cryptoxanthin treatment groups, mice were subjected to intragastric administration of β-cryptoxanthin at 1 mg/kg/day for 3, 7, or 14 days before irradiation, whereas in the control group, mice were treated with the equal volume of corn oil. In the irradiated groups, mice were anesthetized and exposed to a single abdominal irradiation (ABI) of 17 Gy at a dose rate of 0.85 Gy/min using a ^137^Cesium γ-ray irradiator (Atomic Energy of Canadian Inc., Canada). The source-target distance was 30 cm, with the non-targeted body regions shielded by a lead apparatus. Mouse weights were measured every day. On the 3.5th day after irradiation, blood samples were collected, and then animals were sacrificed via euthanasia. Spleens, small intestines, and colons were collected and snap-frozen in liquid nitrogen for qPCR and Western blotting or fixed with 10% formaldehyde for histological assays. The samples of colonic contents were collected when mice were killed. IL-22 in blood serum of mice was measured via the Mouse IL-22 ELISA Kit (R&D, USA), according to the manufacturer's instructions. Apoptosis was determined using TUNEL Apoptosis Assay kit (Beyotime, China).

NRF2-deficient (*Nrf2*^-/-^) male mice (on C57BL/6J background, provided by Thomas W. Kensler from the University of Pittsburgh) and littermate wild type controls, 6-8 weeks old (weighing 18-20 g), were maintained in a certified SPF facility at the IRM. The irradiated mice were randomly distributed into four groups as follows: (1) IR+WT; (2) IR+WT+β-cryptoxanthin; (3) IR+*Nrf2*^-/-^; and (4) IR+*Nrf2*^-/-^+β-cryptoxanthin, with five mice per group. The unirradiated mice were randomly distributed into four groups as follows: (1) WT; (2) WT+β-cryptoxanthin; (3) *Nrf2*^-/-^; and (4) *Nrf2*^-/-^+β-cryptoxanthin, with four mice per group. β-Cryptoxanthin treatments and ABI methods were referred to above for C57BL/6J mice. Mouse weights were measured every day. On the 3.5th day after irradiation, the animals were sacrificed via euthanasia. Small intestine and colon length, spleen index, and thymus index were measured. Small intestines and colons were collected and fixed with 10% formaldehyde for histological assays. The villus length and crypt depth were measured in duodenums and jejunums.

### Fecal Microbiota Transplantation (FMT)

Recipient mice (male C57BL/6, 6-8 weeks old) received an antibiotic cocktail (1 g/L ampicillin, 1 g/L neomycin, 1 g/L metronidazole, and 0.5 g/L vancomycin) in their drinking water for 14 days to deplete their gut microbiota. Donor mice (male C57BL/6, 6-8 weeks old) were pretreated with β-cryptoxanthin or the vehicle (corn oil) for 14 days and then subjected to 17 Gy ABI. Fecal suspension preparation and transplantation commenced daily post-irradiation. Fresh fecal pellets were collected and homogenized in sterile PBS (200 mg feces in 2 mL). After intermittent vortexing to form a homogeneous suspension and centrifugation at 500 g for 5 min, the supernatant was collected. Recipient mice received 200 µL of this supernatant via daily oral gavage for 10 consecutive days. Following the FMT period, recipient mice were subjected to 17 Gy ABI, and euthanized on the 3.5th day post-irradiation. The spleen and thymus were excised and weighed, and the lengths of the small intestines and colons were measured. Tissue sections from the duodenum, jejunum, and colon were collected for histopathological analysis.

### Histopathological analysis

The duodenum, jejunum and colon samples were fixed with 10% formaldehyde and embedded in paraffin. The paraffin-embedded tissues were cut into 6 μm sections, fixed on glass slides, and baked at 60 °C for 60 min. The tissue sections were deparaffinized, rehydrated, and stained with hematoxylin and eosin (H&E), alcian blue-periodic acid Schiff (AB-PAS), or immunohistochemical (IHC) reagents. For H&E staining, hematoxylin (Solarbio, China) stained the nucleus and eosin (Solarbio, China) stained the cytoplasm. Villus length and crypt depth were measured in more than five mice per group. For AB-PAS staining, mucus and goblet cells were immunostained using the AB-PAS staining kit (Solarbio, China) according to the manufacturer's protocol. For IHC analysis, after antigen retrieval and endogenous peroxidase blocking, tissue sections were incubated at 4 °C overnight with the primary antibodies. Afterwards, tissue sections were incubated for 1 h with respective secondary antibody, followed by staining with diaminobenzidine (DAB) and hematoxylin. Cell proliferation and intestinal stem cells in duodenum were detected with the antibody against Ki67 (abcam, ab15580) and OLFM4 (CST, #39141), respectively. The images were captured using an Olympus microscope (BX63, Olympus Life Science, China).

### Microbiota 16S rRNA gene sequencing

Feces were collected from colonic contents of mice on the 3.5th day after irradiation, snap-frozen in liquid nitrogen and stored at -80°C. Bacterial DNA from fecal samples was isolated using a QIAamp Fast DNA Stool Mini Kit (Qiagen, Germany). The V4 variable regions of the 16S rRNA gene were amplified using the primers 515F and 806R, purified with Qiagen Gel Extraction Kit (Qiagen, Germany), and sequenced by the Novogene Company (China) on the Illumina platform.

### Untargeted metabolomic analysis

Fecal samples in each group were collected, snap-frozen in liquid nitrogen and stored at -80 °C until analysis. The samples were ground with liquid nitrogen, resuspended with prechilled 80% methanol, and incubated on ice for 5 min. The supernatant was collected by centrifugation at 15,000 g for 20 min at 4 °C and diluted to the final concentration containing 53% methanol by LC-MS grade water. The samples were subsequently transferred to a fresh tube and centrifuged at 15,000 g for 20 min at 4 °C. Finally, the supernatant was subjected to the LC-MS/MS and untargeted metabolomic analysis conducted by Novogene Company (China).

### Quantification of short chain fatty acids (SCFAs)

Fecal samples were resuspended in 900 μL of 0.5% phosphoric acid, mixed by vortexing and then centrifuged at 14,000 g for 10 min. The supernatant (800 μL) was mixed with the equal volume of ethyl acetate, vortexed thoroughly, followed by centrifugation at 14,000 g for 10 min to separate into two phases. The upper organic phase of 600 μL was collected and mixed with 400 μM 4-methylvalerate acid (an internal standard). Samples were analyzed by a 7890B gas chromatography (GC) System (Agilent, USA) coupled with a 5977B MSD mass selective detector (Agilent, USA) on a DB-FFAP capillary column (30 m × 0.25 mm× 0.25 μm; Agilent, USA). The GC oven was programmed as follows: the starting temperature of 90 °C was increased to 160 °C at a rate of 10 °C/min, increased to 240 °C at a rate of 40 °C/min, and finally held for 5 min. Helium was used as the carrier gas with a constant flow rate of 1 mL/min.

### Statistical analysis

Data are displayed as means ± standard deviations and analyzed using GraphPad Prism 8.0 statistical software. Two-tailed Student's *t*-test was used to compare differences between groups. Values of *P* < 0.05 were considered statistically significant. All experiments were performed at least three times.

## Results

### β-Cryptoxanthin mitigates RIII in mice

**Figure 1:**
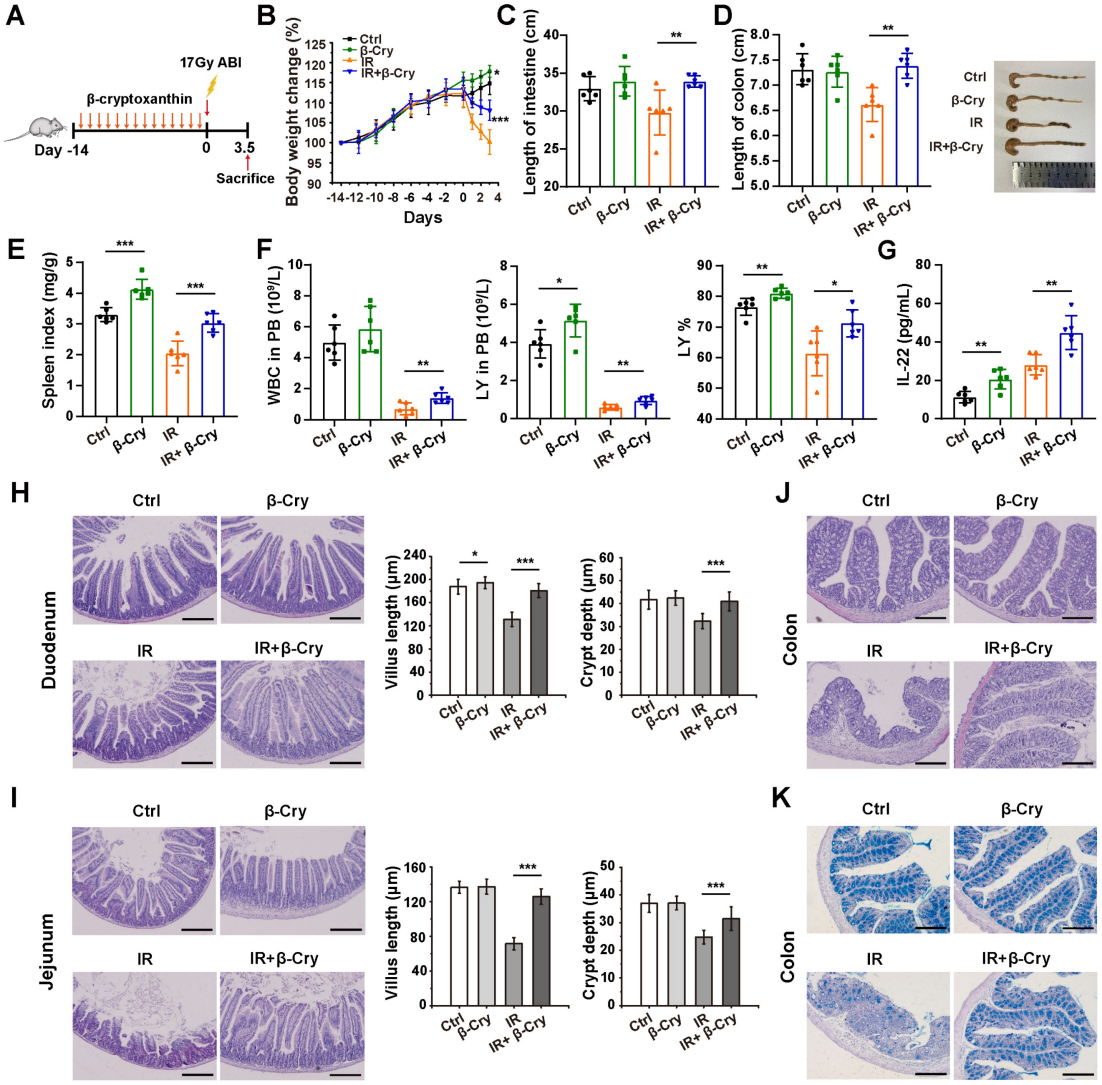
**β-Cryptoxanthin alleviates radiation-induced intestinal injury in mice.** (A) Overview of the experimental scheme. Mice (6 per group) were treated with corn oil (Ctrl) or 1 mg/kg/day β-cryptoxanthin for 14 days and then exposed to 17 Gy ABI. Mice were sacrificed 3.5 day after irradiation. Data are means ± SD. (*n* = 6). (B) Body weights of mice in different treatment groups over time. (C) Length of the small intestines. (D) Length of colons and representative image of colons from mice across the different treatment groups on day 3.5 after 17 Gy ABI. All data are represented as the mean ± SD. (E) Spleen index in different groups. (F) White blood cell (WBC) counts, lymphocytes (LY), and percentage of lymphocytes (LY%) in peripheral blood (PB). Data were presented as means ± SD (*n* = 6 per group). (G) ELISA analysis of IL-22 concentration in the serum of mice. (H-I) H&E staining of duodenum and jejunum sections from mice. Mice were killed and duodenum and jejunum were collected at day 3.5th post irradiation. The scale bar represents 100 μm. Intestinal villus length and crypt depth were measured and shown at the right panel. (J) H&E staining of colon sections from mice. The scale bar represents 100 μm. (K) AB-PAS staining of mouse colon. The acidic mucins in goblet cells were stained blue. The scale bar represents 100 μm. The data are representative of three independent experiments. β-Cry, β-cryptoxanthin.

To evaluate the radioprotective effect of β-cryptoxanthin *in vivo*, C57BL/6 mice were pretreated with β-cryptoxanthin by intragastric administration for 14 days before 17 Gy abdominal irradiation (ABI), and then sacrificed 3.5 days post-irradiation (Fig. 1A). The body weights of mice treated with β-cryptoxanthin increased significantly over time compared with the vehicle-treated controls (Fig. 1B). Irradiated mice consistently lost weight after irradiation, whereas pretreatment with β-cryptoxanthin before irradiation attenuated irradiation-induced weight loss (Fig. 1B). While β-cryptoxanthin alone did not affect the lengths of small intestines and colons in non-irradiated mice, it preserved small intestinal and colonic length against radiation-induced shortening compared to irradiated controls (Fig. 1C-D), suggesting the radioprotective effects of β-cryptoxanthin against RIII. In addition, treatment with β-cryptoxanthin significantly increased the spleen index (Fig. 1E), elevated white blood cell (WBC) counts, lymphocytes (LY), and the percentage of lymphocytes (LY%) in peripheral blood (Fig. 1F), and enhanced serum anti-inflammatory cytokine IL-22 levels (Fig. 1G), suggesting potential immunomodulatory and anti-inflammatory effects against radiation-induced injury.

To further validate the radioprotective role of β-cryptoxanthin in RIII, we examined histological changes in mouse small intestines and colons 3.5 days after 17 Gy ABI. H&E staining analysis revealed that radiation caused crypt and villus structural damage with significant reductions in villus length and crypt depth in the duodenum and jejunum to non-irradiated controls (Fig. 1H-I). β-Cryptoxanthin-pretreated mice showed attenuated histopathological damage, increased crypt survival, and and preserved villus architecture (Fig. 1H-I), further confirming protection against ABI-induced intestinal injury. Immunohistochemical detection of Ki67 and OLFM4 was performed to evaluate cell proliferation and intestinal stem cell population in the duodenum. β-Cryptoxanthin pretreatment before radiation significantly increased Ki67-positive and OLFM4-positive cells in irradiated mice, suggesting enhanced proliferative capacity and stem cell maintenance (Fig. S1A). In colons, radiation induced apparent inflammatory cell infiltrations and epithelial injuries in irradiated mice, which was significantly reduced in mice pretreated with β-cryptoxanthin, suggesting that β-cryptoxanthin pretreatment improved outcomes of radiation-induced colon injury (Fig. 1J). Alcian blue-periodic acid Schiff (AB-PAS) staining analysis revealed that the number of goblet cells was markedly decreased in mice at the 3.5th day after ABI, whereas administration of β-cryptoxanthin effectively attenuated the decrease of goblet cell quantity in the colons (Fig. 1K). To evaluate the effect of β-cryptoxanthin on IR-induced apoptosis, apoptotic cells in the duodenum, jejunum, and colon were detected by TUNEL assays. There were obvious increases of apoptotic cells in all three organs following radiation exposure relative to the control mice. However, pretreatment with β-cryptoxanthin markedly reduced apoptosis levels in the small intestines and colons of irradiated mice (Fig. S1B). Altogether, these results indicate β-cryptoxanthin mitigates RIII by promoting proliferation and regeneration of intestinal cells as well as reducing apoptosis following ABI.

To determine the optimal pretreatment duration, we compared the efficacy of pretreatment with β-cryptoxanthin for 3, 7, or 14 days prior to 17 Gy ABI (Fig. S2A). A 3-day pretreatment did not provide significant protection across systemic and intestinal parameters, including body weight loss, spleen index, and intestinal shortening (Fig. S2B-F). In contrast, both 7-day and 14-day pretreatments significantly mitigated these radiation-induced injuries (Fig. S2B-F). Notably, the 14-day regimen was more effective than the 7-day regimen, particularly in alleviating body weight loss and preserving intestinal architecture (Fig. S2B and Fig. S2G-J). Taken together, these data establish 14 days as the optimal pretreatment duration for β-cryptoxanthin to exert superior radioprotection, highlighting the critical importance of a sufficient pretreatment period.

### The NRF2-mediated antioxidant pathway regulates the protective effect against RIII of β-cryptoxanthin

**Figure 2:**
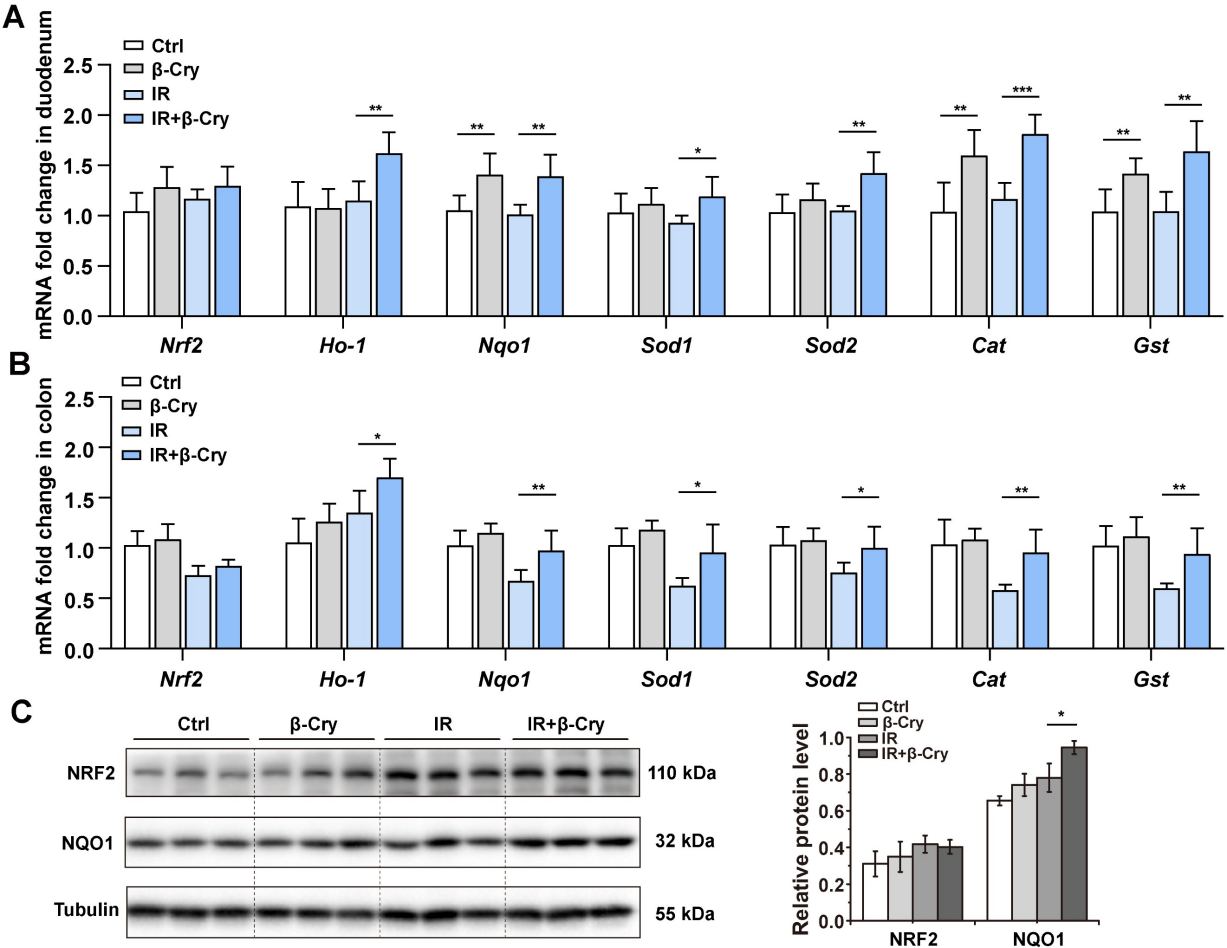
** The protective effect of β-cryptoxanthin against radiation-induced intestinal injury is regulated by the NRF2 antioxidant pathway.** (A) qPCR analysis of *Nrf2* and its downstream target genes expression in mouse duodenums among different groups. Data were presented as means ± SD (*n* = 6 per group). (B) qPCR analysis of *Nrf2* and its downstream target genes expression in mouse colons among different groups. Data were presented as means ± SD (*n* = 6 per group). (C) Western blotting of NRF2 and NQO1 proteins in mouse colons among different groups. The data are representative of three independent experiments. β-Cry, β-cryptoxanthin.

The transcription factor NRF2 plays a pivotal role in the radioprotective response by coordinating the expression of key antioxidant enzymes 29. To investigate the correlation between the NRF2-mediated antioxidant pathway and β-cryptoxanthin's radioprotective effects in mice, mRNA levels of *Nrf2* and its downstream target genes were analyzed through qPCR. Pretreatment with β-cryptoxanthin significantly increased the transcription of *Ho-1*, *Nqo1*, *Sod1*, *Sod2*, *Cat*, and *Gst* genes in the duodenum of the irradiated mice (Fig. 2A), with similar results in colon and spleen (Fig. 2B and Fig. S3). Additionally, β-cryptoxanthin elevated NQO1 protein level in colons of the irradiated mice (Fig. 2C).

**Figure 3:**
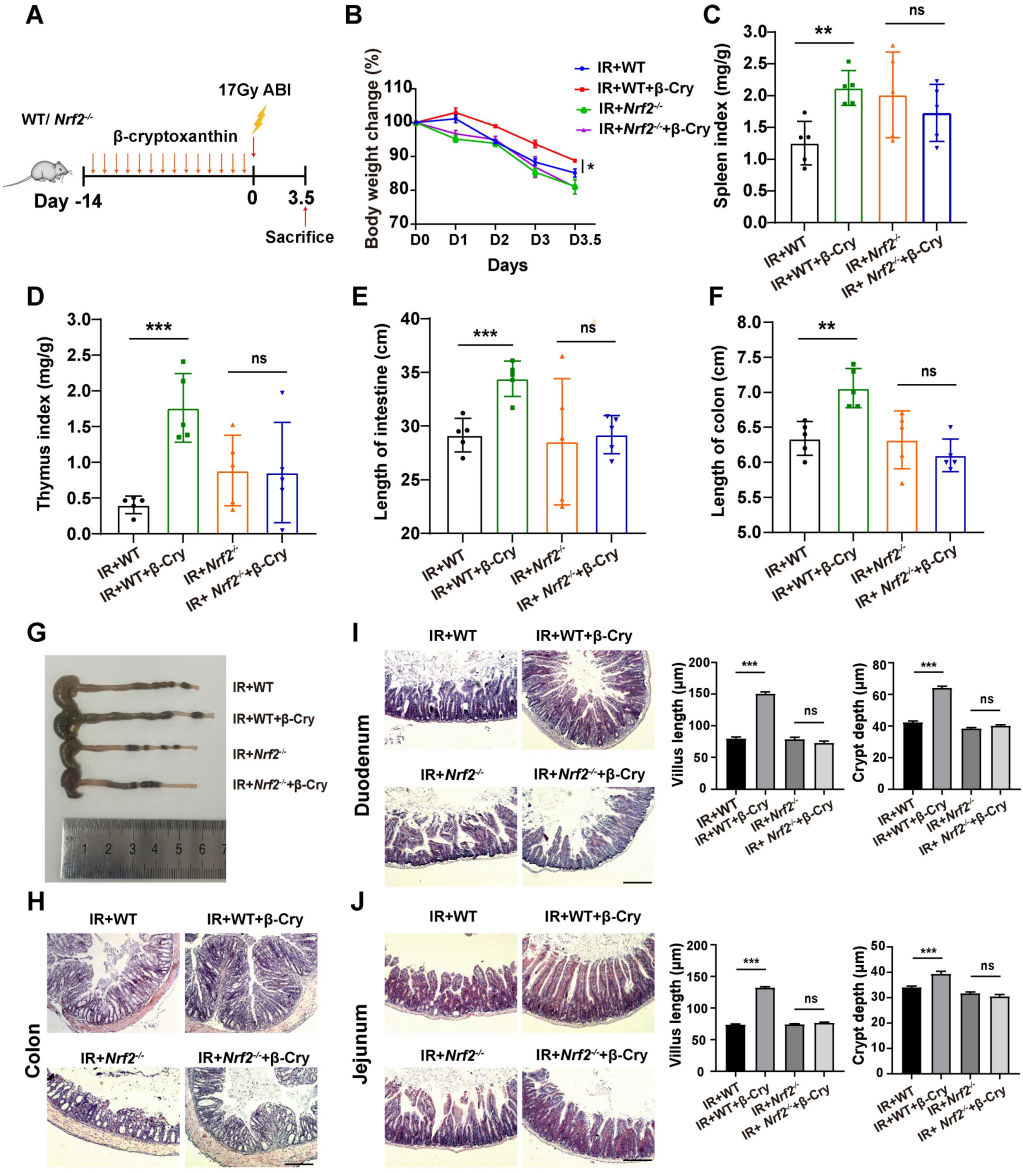
** β-Cryptoxanthin failed to mitigate radiation-induced intestinal injury in *Nrf2*^-/-^ mice.** (A) Overview of the experimental scheme. Mice (5 per group) were treated with corn oil (Ctrl) or 1 mg/kg/day β-cryptoxanthin for 14 days and then exposed to 17 Gy ABI. Mice were sacrificed on the 3.5th day. Data are means ± SD. (n = 5). (B) Body weights of mice in different treatment groups over time. (C) Spleen index in different groups. (D) Thymus index in different groups. (E) Length of the small intestines. (F-G) Length of colons and representative image of colons from mice across the different treatment groups on day 3.5 after 17 Gy ABI. (H) H&E staining of colon sections from mice. The scale bar represents 100 μm. (I-J) H&E staining of duodenum and jejunum sections from mice. The scale bar represents 100 μm. Intestinal villus length and crypt depth were measured and shown at the right panel. β-Cry, β-cryptoxanthin.

To further evaluate the effect of NRF2 on the radioprotection of β-cryptoxanthin to mice, we also conducted animal experiments using wild-type (WT) and *Nrf2*-knockout (*Nrf2*^-/-^) mice (Fig. 3A and Fig. S4A). β-Cryptoxanthin pretreatment did not affect body weight (Fig. S4B), spleen and thymus indices (Fig. S4C-D), small intestine and colon lengths (Fig. S4E-G), or histopathology in non-irradiated mice of either genotype (Fig. S4H-J). Following irradiation, β-cryptoxanthin significantly ameliorated weight loss (Fig. 3B), preserved spleen/thymus indices (Fig. 3C-D), maintained small intestine and colon lengths (Fig. 3E-G), and reduced RIII severity in WT mice (Fig. 3H-J). Conversely, it failed to mitigate RIII in *Nrf2*^-/-^ mice (Fig. 3E-J), indicating NRF2-dependency. Collectively, β-cryptoxanthin alleviates RIII by promoting proliferation/regeneration and suppressing apoptosis through the NRF2-mediated antioxidant pathway.

### β-Cryptoxanthin has antioxidant and radioprotective effects on MODE-K cells

To investigate the cellular level of radioprotective effect, different concentrations of β-cryptoxanthin were administered to the mouse intestinal epithelial cell line MODE-K and the human intestinal epithelial cell line HIEC-6 to evaluate its toxicity. β-Cryptoxanthin exhibited no significant cytotoxicity (as assessed by cell viability) at concentrations below 40 μM after 48 h of treatment via CCK-8 assays (Fig. S5). Additionally, concentrations below 20 μM showed no significant effect on the proliferation of MODE-K and HIEC-6 cells compared to untreated controls (Fig. S5). Hence, concentrations of 5, 10, and 20 μM were selected for subsequent bioassays.

**Figure 4:**
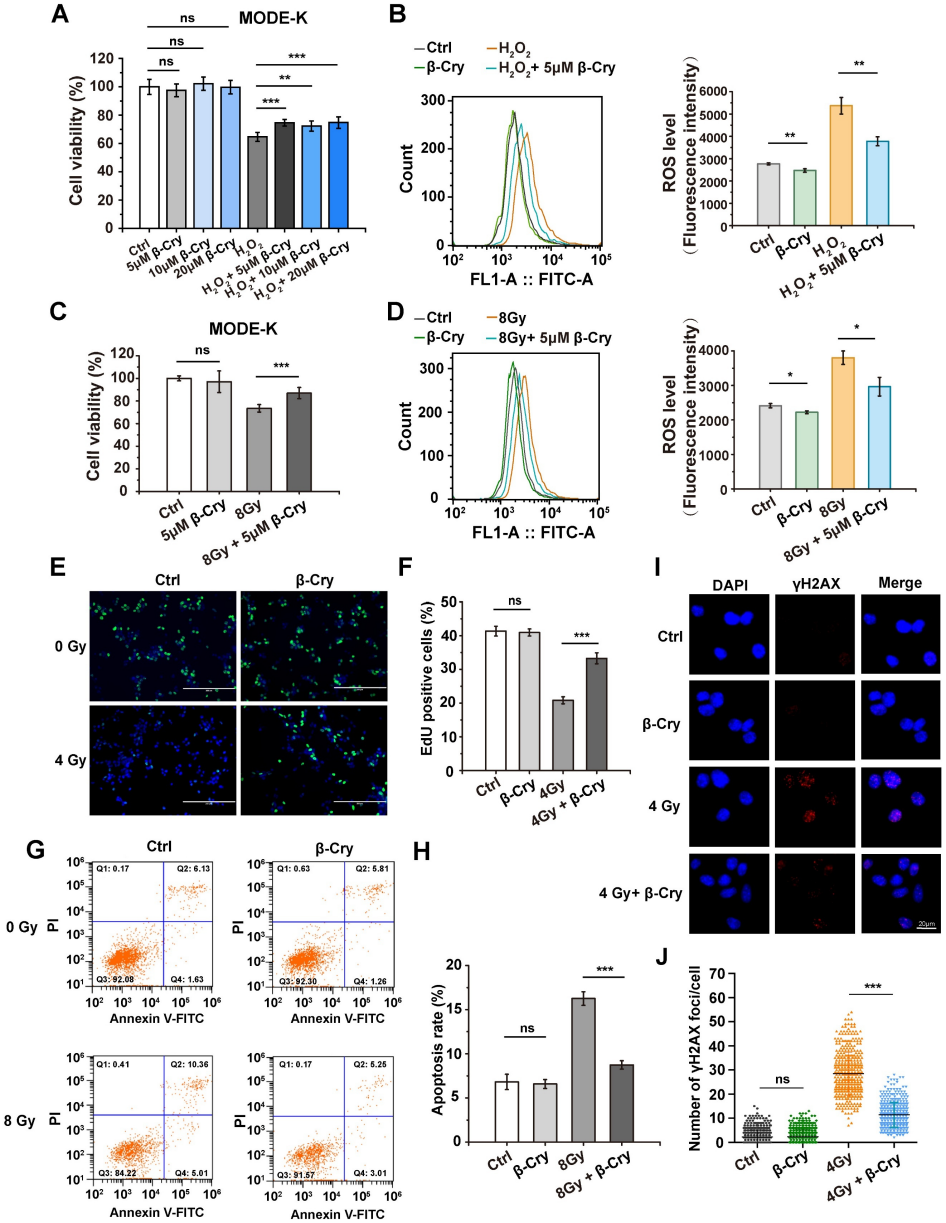
** β-Cryptoxanthin has antioxidant and radioprotective effects on MODE-K cells.** (A) Effect of β-cryptoxanthin (5, 10, 20 μM) pretreatment on cell survival after hydrogen peroxide (400 μM) treatment of MODE-K cells. (B) ROS levels in MODE-K cells at 1 h after hydrogen peroxide (400 μM) treatment. (C) Effect of β-cryptoxanthin pretreatment on cell survival after radiation treatment of MODE-K cells. (D) ROS levels in MODE-K cells at 1 h after radiation treatment. (E) Representative images of EdU staining in MODE-K cells in different groups. EdU positive cells are green and nuclei are counterstained with DAPI (blue). Scale bars, 200 μm. (F) Quantitative analysis of percentage of EdU-positive cells. (G) The apoptosis levels of MODE-K cells measured by flow cytometry. Cells were pretreated with β-cryptoxanthin and then exposed to 8 Gy IR. The apoptosis of cells was assessed by co-staining with FITC-Annexin V and PI at 48 h post-irradiation. (H) Quantitative analysis of apoptosis rates of MODE-K cells at 48 h post-irradiation. Data are means ± SD from three independent experiments. (I) Representative images of immunofluorescence staining for γH2AX foci (red dots). MODE-K cells were pretreated with β-cryptoxanthin for 12 h and then exposed to 4 Gy IR. γH2AX foci were counted at 6 h post-irradiation. Scale bars, 20 μm. (J) Quantitative analysis of γH2AX foci (red) numbers per cell. Data are means ± SD, with more than 150 cells were counted from three independent experiments. β-Cry, β-cryptoxanthin.

To investigate the antioxidant effect of β-cryptoxanthin, MODE-K cells were pretreated with 5, 10, or 20 μM β-cryptoxanthin for 12 h before hydrogen peroxide (400 μM) treatment. Pretreatment with β-cryptoxanthin at 5, 10, or 20 μM significantly increased the viability of MODE-K cells compared to cells exposed to hydrogen peroxide alone, with no statistically significant differences among these concentrations (Fig. 4A). Based on the principle of selecting the lowest effective concentration, the concentration of 5 μM was selected for subsequent experiments. Furthermore, β-Cryptoxanthin at 5 μM significantly decreased intracellular ROS levels in MODE-K cells as compared to cells treated with hydrogen peroxide alone (Fig. 4B), implying that β-cryptoxanthin is able to protect cells against hydrogen peroxide-induced cytotoxicity and inhibit hydrogen peroxide-induced oxidative stress.

Radiation induces oxidative stress and cell damage through ROS generation 7. Similar to its effects against hydrogen peroxide, β-cryptoxanthin pretreatment significantly increased viability (Fig. 4C) and reduced ROS levels (Fig. 4D) in MODE-K cells exposed to 8 Gy γ-irradiation compared to untreated irradiated cells, indicating that β-cryptoxanthin exerts a radioprotective effect on MODE-K cells by attenuating radiation-induced oxidative stress through ROS scavenging. To test the generality of this effect, we evaluated β-cryptoxanthin in other cell types. Indeed, it also increased the viability of HIEC-6 cells treated with either hydrogen peroxide or radiation (Fig. S6A-B). Moreover, β-cryptoxanthin significantly enhanced the viability of both MRC-5 and HK-2 cells after 8 Gy irradiation (Fig. S6C-D), indicating its broad-spectrum radioprotective potential. EdU staining was used to assess cell proliferation, which showed that β-cryptoxanthin pretreatment increased the proportion of proliferating (EdU-positive) MODE-K cells after irradiation (Fig. 4E-F), demonstrating its role in preserving proliferative capacity under oxidative stress caused by radiation.

Radiation induces cell apoptosis, DNA damage, and cell cycle arrest 30. To evaluate the anti-apoptotic effect of β-cryptoxanthin, apoptosis in MODE-K cells was assessed by flow cytometry after pretreatment with β-cryptoxanthin followed by radiation exposure. β-Cryptoxanthin pretreatment significantly reduced radiation-induced apoptosis (by 46%, P< 0.001) in cells exposed to 8 Gy (Fig. 4G-H). DNA damage was quantified via γH2AX foci formation, as γH2AX can recruit repair proteins to DNA damage sites in the initial step of the DNA damage response 31. The irradiated MODE-K cells had a remarkable increase in γH2AX foci compared with the control group, whereas β-cryptoxanthin pretreatment significantly reduced γH2AX foci at 6 h and 12 h after exposure to 4 Gy radiation (Fig. 4I-J and Fig. S7), suggesting that β-cryptoxanthin could reduce DNA damage induced by irradiation. Notably, β-cryptoxanthin pretreatment did not significantly alter the cell cycle distribution in irradiated MODE-K cells (Fig. S8), indicating that its radioprotective mechanism is independent of alleviating radiation-induced cell cycle arrest. Collectively, β-cryptoxanthin protects MODE-K cells against hydrogen peroxide and radiation-induced cell damage by reducing ROS level, promoting proliferation, decreasing apoptosis, and reducing DNA damage.

### β-Cryptoxanthin enhances the NRF2-mediated antioxidant pathway in MODE-K cells

**Figure 5:**
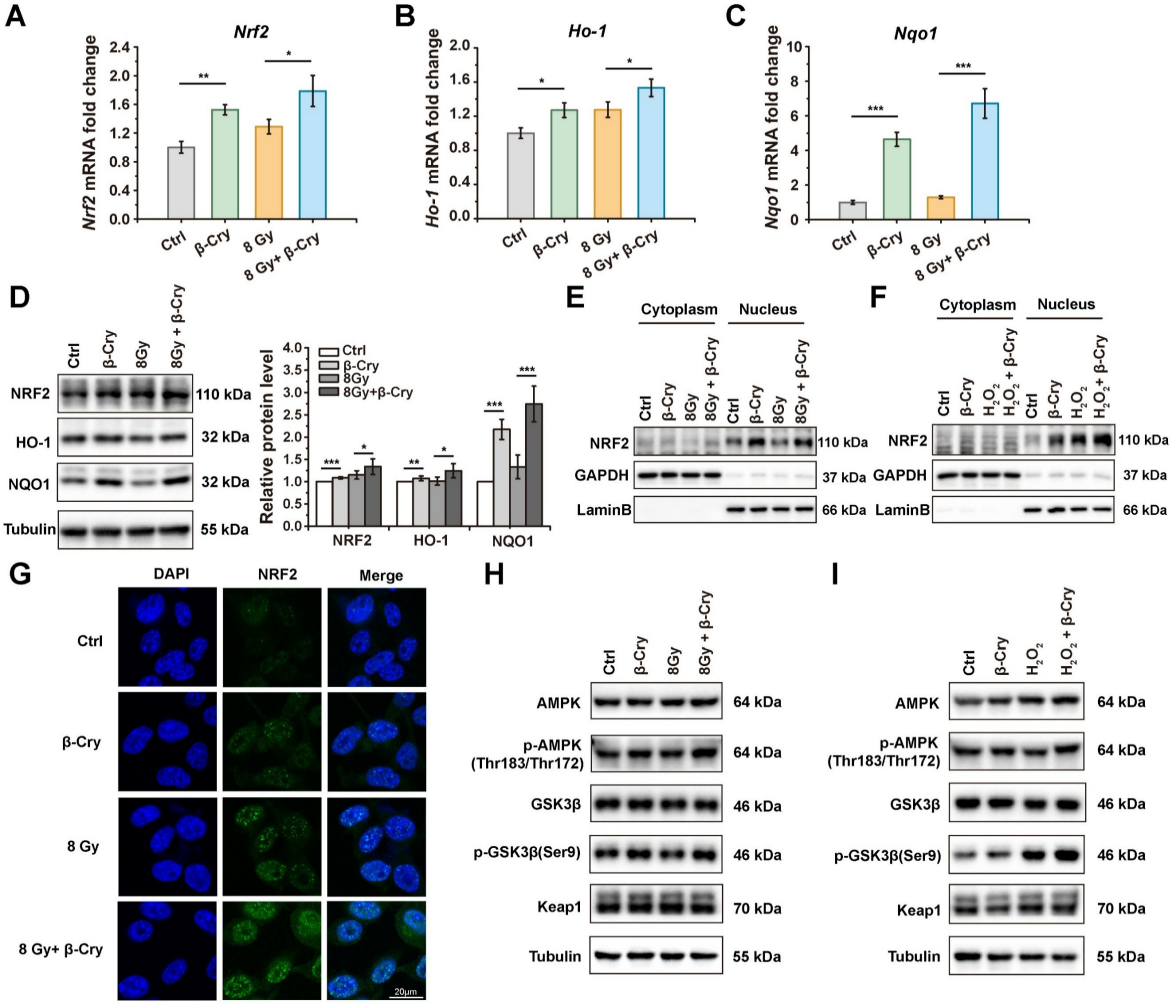
** β-Cryptoxanthin enhances the NRF2-mediated antioxidant pathway in MODE-K cells.** (A-C) The mRNA levels of *Nrf2*, *Ho-1*, and *Nqo1* in MODE-K cells were determined by qPCR. Cells were pretreated with β-cryptoxanthin before exposure to 8 Gy IR and harvested at 8 h after IR. The results are the mean ± SD of three experiments, each in triplicate. (D) Western blotting of NRF2, HO-1 and NQO1 in MODE-K cells pretreated with β-cryptoxanthin and then exposed to 8 Gy IR. Cells were harvested at 8 h after IR. Tubulin was a loading control. Quantitative analysis of expression of the proteins was conducted using Image Lab software (version 6.1, Bio-rad). The data are representative of three independent experiments. (E) Western blotting of NRF2 in the cytoplasm and nucleus in MODE-K cells pretreated with β-cryptoxanthin and exposed to 8 Gy IR. (F) Western blotting of NRF2 in the cytoplasm and nucleus in MODE-K cells pretreated with β-cryptoxanthin and then exposed to hydrogen peroxide. (G) The immunofluorescence analysis of NRF2 distribution in MODE-K cells at 8 h post-irradiation. Scale bar, 20 μm. (H-I) Effects of β-cryptoxanthin on expression of proteins related to nucleus translocation of NRF2 as detected by Western blotting. MODE-K cells were pretreated with β-cryptoxanthin for 12 h and then exposed to 8 Gy IR (H) or hydrogen peroxide (I). β-Cry, β-cryptoxanthin.

To investigate the cellular level of radioprotective mechanism, NRF2 and its downstream target genes *Ho-1* and *Nqo1* encoding for heme oxygenase-1 (HO-1) and NAD(P)H:quinone oxidoreductase 1 (NQO1) were analyzed in mRNA levels via quantitative PCR (qPCR) and protein levels via Western blotting. Compared to the β-cryptoxanthin-untreated group, treatment with β-cryptoxanthin significantly upregulated the mRNA levels of *Nrf2*, *Ho-1*, and *Nqo1* (Fig. 5A-C), whether irradiated or not. The variation tendency of the protein levels of NRF2, HO-1, and NQO1 was similar to that of mRNA levels, in which β-cryptoxanthin significantly upregulated the protein level of NQO1 (Fig. 5D). Consistent with the results of irradiation, pretreatment with β-cryptoxanthin before exposure to hydrogen peroxide also upregulated the mRNA level of *Nrf2* and its downstream target genes (*Ho-1* and *Nqo1*) (Fig. S9A-C), as well as the levels of the corresponding proteins (Fig. S9D). These data indicated that β-cryptoxanthin enhanced the NRF2-mediated antioxidant pathway by activating *Nrf2* and its downstream target genes in MODE-K cells, thereby providing a potential mechanism underlying its radioprotective effect.

NRF2 can translocate into the nucleus upon activation, thereby triggering the transcription of its downstream antioxidant targets 32. To test whether β-cryptoxanthin could promote the nuclear translocation of NRF2 in MODE-K cells, cytoplasmic and nuclear proteins were isolated and NRF2 in these fractions was detected by Western blotting. When MODE-K cells were pretreated with β-cryptoxanthin before exposure to radiation or hydrogen peroxide, the NRF2 levels accumulated in the nucleus (Fig. 5E-F), indicating that β-cryptoxanthin induced the nuclear translocation of NRF2. Consistently, immunofluorescence assay also demonstrated that pretreatment with β-cryptoxanthin increased the accumulation of NRF2 in the nucleus (Fig. 5G).

To explore the mechanism of β-cryptoxanthin promoting the stabilization and nuclear translocation of NRF2, we performed Western blotting to detect the protein levels of possible upstream mediators. The total protein level of Keap1 did not change significantly by β-cryptoxanthin treatment (Fig. 5H-I), suggesting potential Keap1-independent regulation of NRF2 stabilization and nuclear translocation. Glycogen synthase kinase 3β (GSK3β) promotes nuclear export and suppresses nuclear import of NRF2, while its phosphorylation at serine 9 residue inactivates GSK3β, thus enhancing NRF2 nuclear translocation and activation 33. AMP-activated protein kinase (AMPK) may mediate the inactivation of GSK3β 34. β-Cryptoxanthin pretreatment increased phosphorylation of AMPK and GSK3β (Ser9) compared with irradiation-only or hydrogen peroxide-only treated groups (Fig. 5H-I), indicating AMPK-mediated GSK3β inactivation enhances NRF2 nuclear translocation and activation.

**Figure 6:**
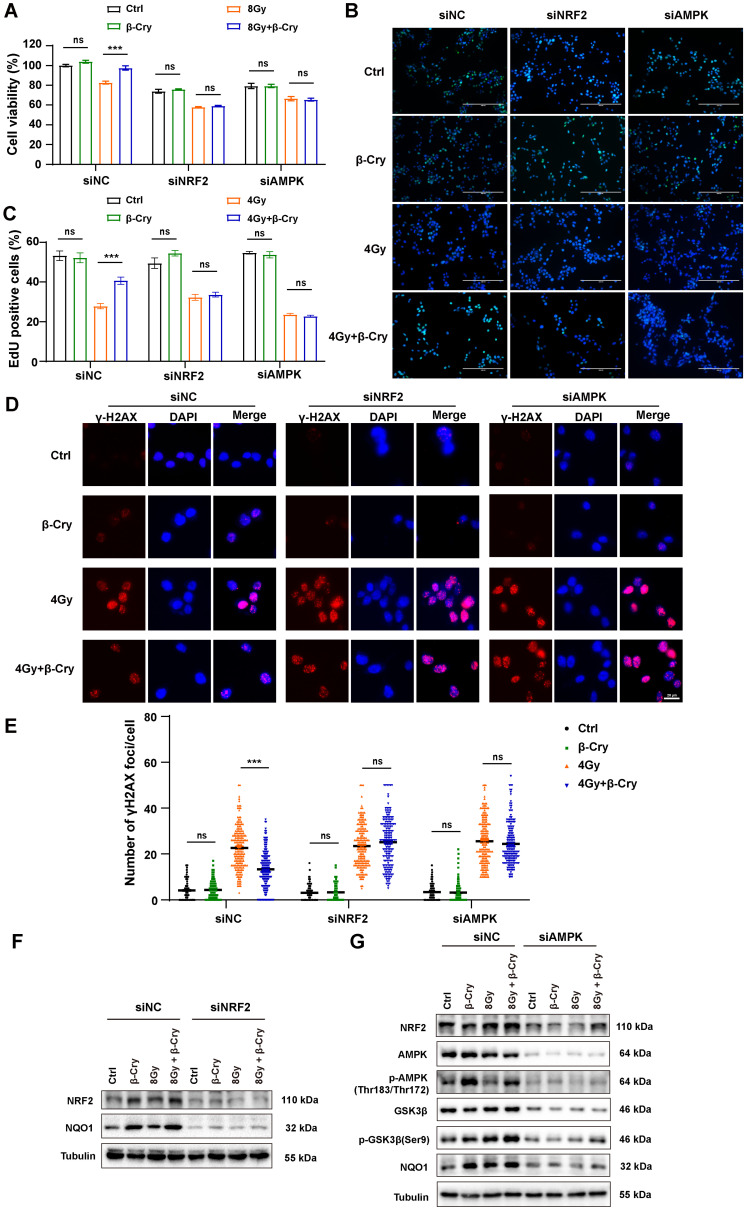
** NRF2 or AMPK knockdown abolished the ability of β-cryptoxanthin to mitigate radiation-induced damage and enhance the NRF2-mediated antioxidant pathway in MODE-K cells.** (A) Effect of β-cryptoxanthin pretreatment on cell survival after radiation treatment of MODE-K cells when NRF2 or AMPK was knocked down. (B) Representative images of EdU staining in MODE-K cells in different groups. EdU positive cells are green and nuclei are counterstained with DAPI (blue). Scale bars, 200 μm. (C) Quantitative analysis of percentage of EdU-positive cells. (D) Representative images of immunofluorescence staining for γH2AX foci (red dots). γH2AX foci were counted at 6 h post-irradiation. Scale bars, 20 μm. (E) Quantitative analysis of γH2AX foci (red) numbers per cell. (F) The protein levels of NRF2 and NQO1 as detected by Western blotting when MODE-K cells were treated with NRF2-targeting siRNA. (G) The protein levels of NRF2, NQO1 and AMPK-GSK3β pathway as detected by Western blotting when MODE-K cells were treated with AMPK-targeting siRNA. Data are means ± SD, with more than 150 cells were counted from three independent experiments. β-Cry, β-cryptoxanthin.

To confirm the central role of NRF2 and AMPK in the radioprotective effects of β-cryptoxanthin, we performed siRNA-mediated knockdown of NRF2 or AMPK in MODE-K cells. The protective effects of β-cryptoxanthin were completely abrogated in NRF2- or AMPK-knockdown cells. Specifically, in NRF2- or AMPK-knockdown cells, β-cryptoxanthin failed to enhance the viability of irradiated cells (Fig. 6A) or promote cell proliferation (assessed via EdU proliferation assay, Fig. 6B-C). Moreover, despite β-cryptoxanthin treatment, NRF2/AMPK-knockdown cells exhibited DNA damage levels comparable to the irradiation-only group (Fig. 6D-E). Critically, β-cryptoxanthin's upregulation of NRF2-mediated antioxidant pathway proteins was suppressed upon knockdown of either NRF2 or AMPK (Fig. 6F-G), and AMPK knockdown reduced p-GSK3β (Ser9) protein levels (Fig. 6G). These results demonstrate that β-cryptoxanthin mitigates radiation-induced damage in MODE-K cells primarily through activation of the AMPK and NRF2 signaling pathways.

### β-Cryptoxanthin reprograms the gut microbiota of irradiated mice

**Figure 7:**
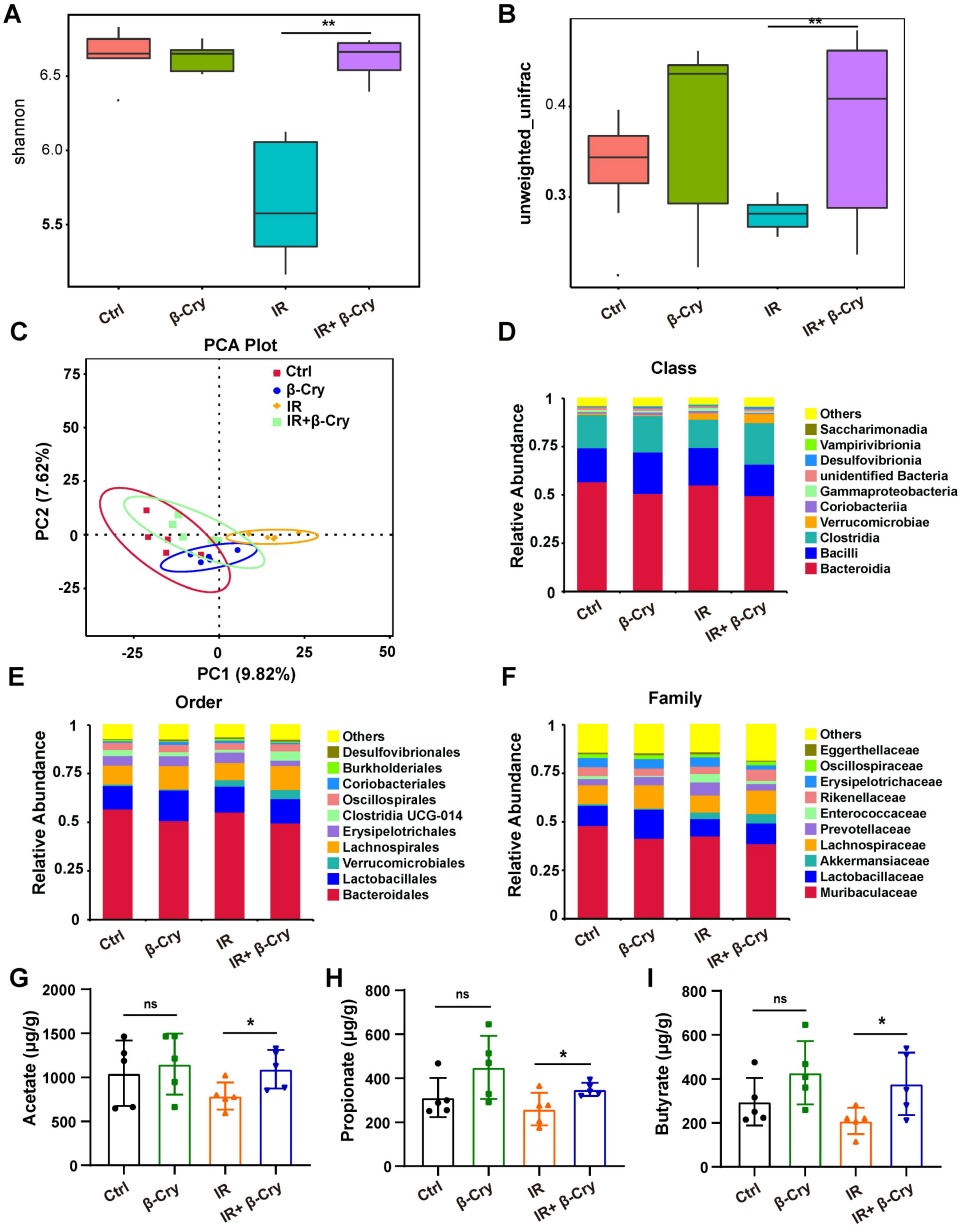
** β-Cryptoxanthin improved the gut microbiota in irradiated mice.** The feces in different groups were collected at 3.5th day after irradiation and analyzed by 16S rRNA gene sequencing. (A) The α diversity of intestinal bacteria from the indicated treatment groups, in which Shannon index represents the community diversity. (B) The β diversity index of intestinal bacteria in different groups based on the unweighted UniFrac distance, assessing the interindividual differences. (C) Principal component analysis (PCA) plot of gut microbiota based on operational taxonomic units (OTUs) analysis in the fecal samples. Axes correspond to the principal components 1 *(x* axis) and 2 (*y* axis). Each dot indicates a single sample. (D) Relative abundance of intestinal bacteria at the class level in fecal samples of mice. *n* = 5 mice per group. (E) Relative abundance of intestinal bacteria at the order level in fecal samples of mice. *n* = 5 mice per group. (F) Relative abundance of intestinal bacteria at the family level in fecal samples of mice. *n* = 5 mice per group. (G-I) Levels of SCFAs are increased in β-Cryptoxanthin-treated mice. Acetate (G), propionate (H), and butyrate (I) levels in the fecal materials were detected by GC-MS. Fecal samples were collected at the 3.5th day post radiation. Data were presented as means ± SD (n = 5 per group). β-Cry, β-cryptoxanthin.

To investigate the impact of β-cryptoxanthin on the intestinal microenvironment, we analyzed gut microbiota composition via 16S rRNA gene sequencing. Diversity analysis revealed that radiation exposure significantly reduced both α-diversity (Shannon index) and β-diversity compared to the unirradiated controls (Fig. 7A-B). However, β-cryptoxanthin pretreatment prior to irradiation markedly improved bacterial diversity in irradiated mice (Fig. 7A-B). Principal component analysis (PCA) further demonstrated a significant shift in gut bacterial structure post-irradiation, while β-cryptoxanthin pretreatment attenuated these alterations, restoring the microbial community composition toward a state resembling unirradiated controls (Fig. 7C). These findings indicate that β-cryptoxanthin ameliorates radiation-induced dysbiosis of gut microbiota by preserving microbial diversity and community structure.

To identify specific microbial taxa affected by β-cryptoxanthin, we performed a Linear Discriminant Analysis Effect Size (LEfSe) analysis of significantly different species among groups (Fig. S10). Notably, the family *Enterococcaceae*, which contains many opportunistic pathogens 35, 36, was identified as a significant biomarker in the IR group (Fig. S10A-B). In contrast, β-cryptoxanthin treatment promoted a distinct microbial signature characterized by the enrichment of SCFA-producing families, including *Lachnospiraceae* and *Rikenellaceae* (Fig. S10C-D) 37, 38. The relative abundance analysis revealed an increased in the abundance of *Clostridia* at the class level in the feces of β-cryptoxanthin-treated mice (Fig. 7D), with corresponding increases in *Lachnospirales* and *Lachnospiraceae*, at the levels of order and family, respectively (Fig. 7E-F). Furthermore, β-cryptoxanthin treatment also increased the abundance of several beneficial bacteria such as *Akkermansiaceae* and *Lactobacillaceae* in irradiated mice (Fig. 7F). SCFAs are important substrates for maintaining the intestinal epithelium and regulating gut immunity and inflammatory response 39. Consistent with the increased abundance of *Rikenellaceae*, *Lachnospiraceae*,* Akkermansiaceae*, and *Lactobacillaceae* - families associated with SCFA production - acetate, propionate, and butyrate levels were significantly elevated in the fecal samples of β-cryptoxanthin-pretreated mice when exposed to ABI (Fig. 7G-I). To directly investigate whether these metabolites contribute to radioprotection, we assessed their effects on intestinal epithelial cells* in vitro*. Pretreatment of MODE-K cells with acetate (5 mM), propionate (1 mM), or butyrate (1 mM) significantly enhanced cell viability after 8 Gy irradiation (Fig. S11), demonstrating that the elevated SCFAs can directly protect intestinal cells from radiation-induced damage. Collectively, β-cryptoxanthin might play a radioprotective role in irradiated mice by restoring the gut microbiota impaired by irradiation through enhancing the abundance of beneficial bacteria and associated protective metabolites such as SCFAs.

**Figure 8:**
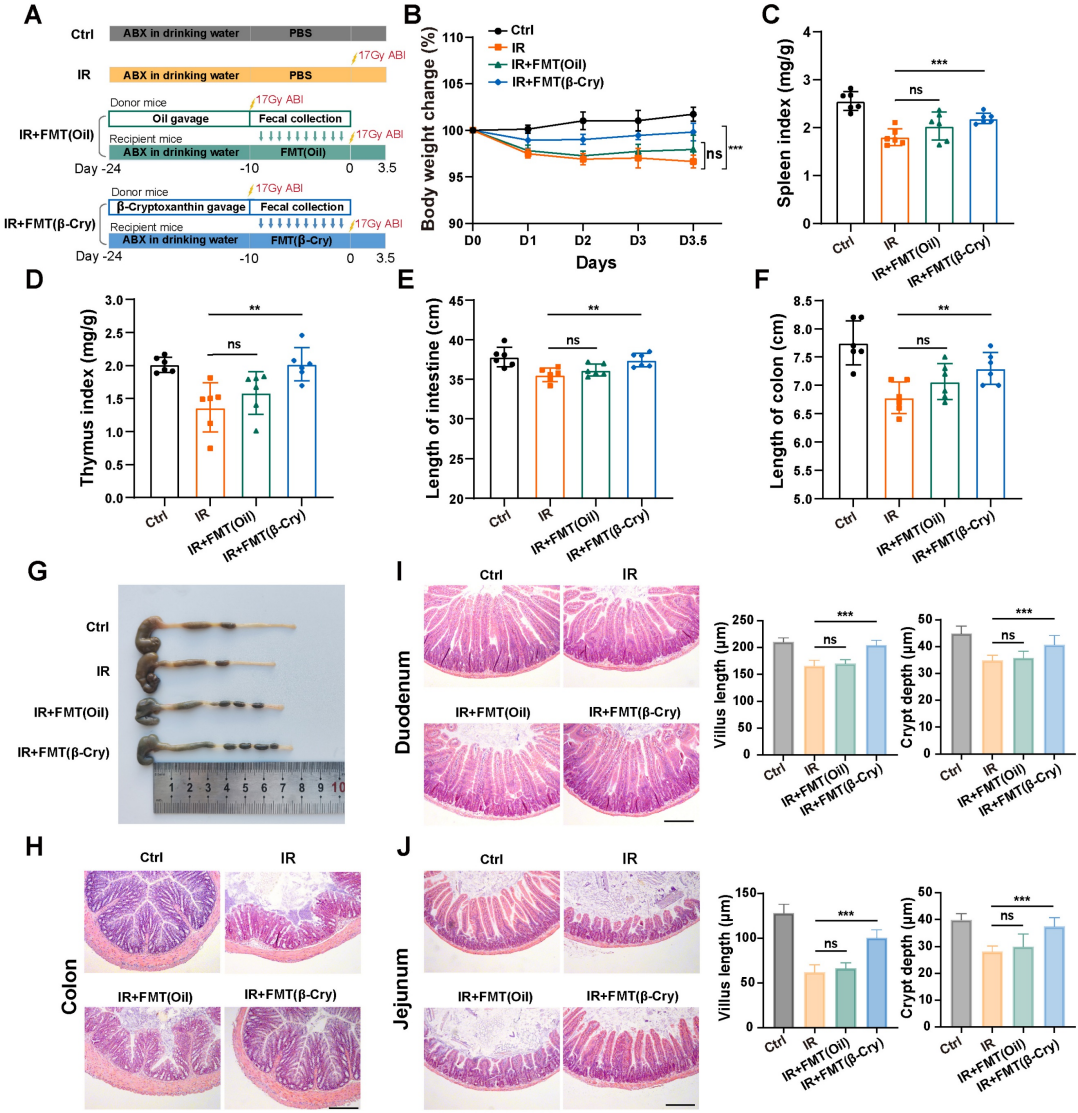
** Fecal microbiota transplantation from β-cryptoxanthin-treated donors confers radioprotection.** (A) Overview of the experimental scheme. Donor mice (6 per group) were pretreated with β-cryptoxanthin or corn oil for 14 days, followed by 17 Gy ABI. Fecal microbiota from these donors was then transplanted into recipient mice that had been pretreated with an antibiotic cocktail for 14 days. Recipient mice (6 per group) were exposed to 17 Gy ABI, and were sacrificed on the 3.5th day. (B) Body weights of mice in different treatment groups over time. (C) Spleen index in different groups. (D) Thymus index in different groups. (E) Length of the small intestines. (F-G) Length of colons and representative image of colons from mice across the different treatment groups on day 3.5 after 17 Gy ABI. (H) H&E staining of colon sections from mice. (I) H&E staining of duodenum sections from mice. Intestinal villus length and crypt depth were measured and shown at the right panel. (J) H&E staining of jejunum sections from mice. Intestinal villus length and crypt depth were measured and shown at the right panel. The scale bars represent 100 μm. β-Cry, β-cryptoxanthin.

To directly evaluate whether the gut microbiota reprogrammed by β-cryptoxanthin mediates radioprotection, we performed an FMT experiment. Donor mice were pretreated with β-cryptoxanthin or vehicle (corn oil) for 14 days and then subjected to 17 Gy ABI. The fecal microbiota from these donors was transplanted into recipient mice that had been pretreated with an antibiotic cocktail (Fig. 8A). As shown in Fig. 8, FMT from β-cryptoxanthin-treated donors significantly alleviated systemic and intestinal radiation injury in recipients. This protection was evident in mitigated body weight loss, preserved spleen and thymus indices, and reduced intestinal shortening (Fig. 8B-G). In contrast, FMT from vehicle-treated donors failed to provide significant protection. Histological analysis further demonstrated that transplantation of microbiota from β-cryptoxanthin-treated donors markedly improved intestinal architecture. In the colon, it reduced inflammatory cell infiltration and epithelial damage (Fig. 8H). In the small intestine (duodenum and jejunum), it ameliorated radiation-induced villus shortening and increased crypt depth (Fig. 8I-J). These results indicate that the gut microbiota reprogrammed by β-cryptoxanthin contributes to radioprotection and can mitigate radiation-induced intestinal injury.

### β-Cryptoxanthin alters the intestinal metabolites of irradiated mice

**Figure 9:**
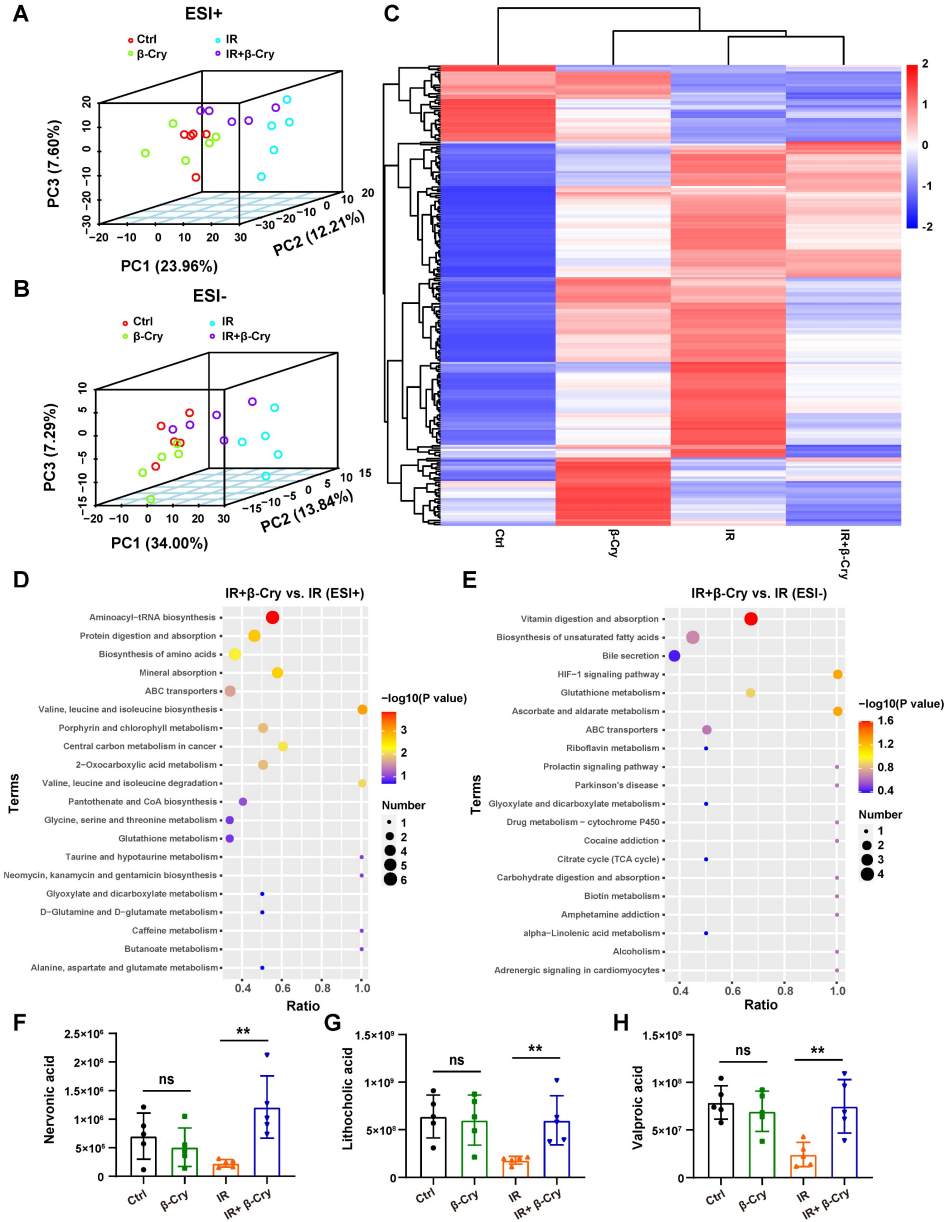
** β-Cryptoxanthin altered the intestinal metabolites in irradiated mice.** (A-B) The 3-dimensional PCA plot of fecal metabolite composition in each group obtained by LC-MS/MS ES+ and ES-. Each data point indicates a single sample and each group is shown in a different color. (C) Heatmap analysis of the differential metabolites in negative ion mode. (D-E) KEGG pathway enrichment analysis when pretreated with β-Cryptoxanthin in the irradiated mice obtained by LC-MS/MS ES+ and ES- (n = 5). (F-H) The relative quantitative analysis of nervonic acid (F), lithocholic acid (G), and valproic acid (H). β-Cry, β-cryptoxanthin.

To investigate whether β-cryptoxanthin's radioprotection involves altered bacterial metabolites, an untargeted metabolomic analysis was performed using fecal samples from mice in different groups. PCA was used to evaluate the group differences in metabolite profiles. The 3-dimensional PCA plot showed that the composition of gut metabolites in the IR group was distinct from the control group, both in positive and negative mode, implying the metabolic perturbations caused by irradiation (Fig. 9A-B). However, mice pretreated with β-cryptoxanthin before irradiation exhibited a metabolite profile closer to that of the unirradiated mice (Fig. 9A-B), indicating that β-cryptoxanthin has protective effects on intestinal metabolic homeostasis in mice. Heat map analysis of differential metabolites revealed that the metabolomic profiles of β-cryptoxanthin-treated groups (irradiated and non-irradiated) were similar to those of the control group, whereas those of the IR group showed the greatest divergence from the control group in the negative ion mode (Fig. 9C).

Through analysis of alterations in metabolites of irradiated mice, a total of 42 and 103 metabolites were revealed to be significantly upregulated in ESI-positive and negative mode, respectively, following pretreatment with β-cryptoxanthin (Fig. S12). KEGG pathway enrichment analysis indicated that β-cryptoxanthin elicited major alterations in metabolic pathways related to aminoacyl-tRNA biosynthesis, protein digestion and absorption, vitamin digestion and absorption, biosynthesis of unsaturated fatty acids and amino acids, and bile secretion (Fig. 9D-E). Relative quantitative analysis of differential metabolites in these KEGG pathways revealed that the levels of nervonic acid, lithocholic acid, and valproic acid were significantly increased in mice treated with β-cryptoxanthin prior to irradiation compared with the irradiation-only group (Fig. 9F-H). Nervonic acid, a long-chain unsaturated fatty acid, promotes cell proliferation and differentiation, which is crucial for maintaining normal tissue growth and repair 40. Furthermore, it exerts significant anti-inflammatory effects by regulating pro-inflammatory signaling pathways and metabolic processes 41. Regarding the bile secretion pathway, an abnormal bile acid profile contributed to RIII, whereas treatment with the secondary bile acid lithocholic acid promoted intestinal recovery from this damage 42. Valproic acid, a potent radioprotector, alleviates total-body irradiation-induced small intestinal mucositis in mice and protects mouse intestinal organoids against radiation 43, 44. These results reveal that β-cryptoxanthin can alter intestinal metabolites and gut metabolic pathways, thus alleviating RIII in mice.

## Discussion

**Figure 10:**
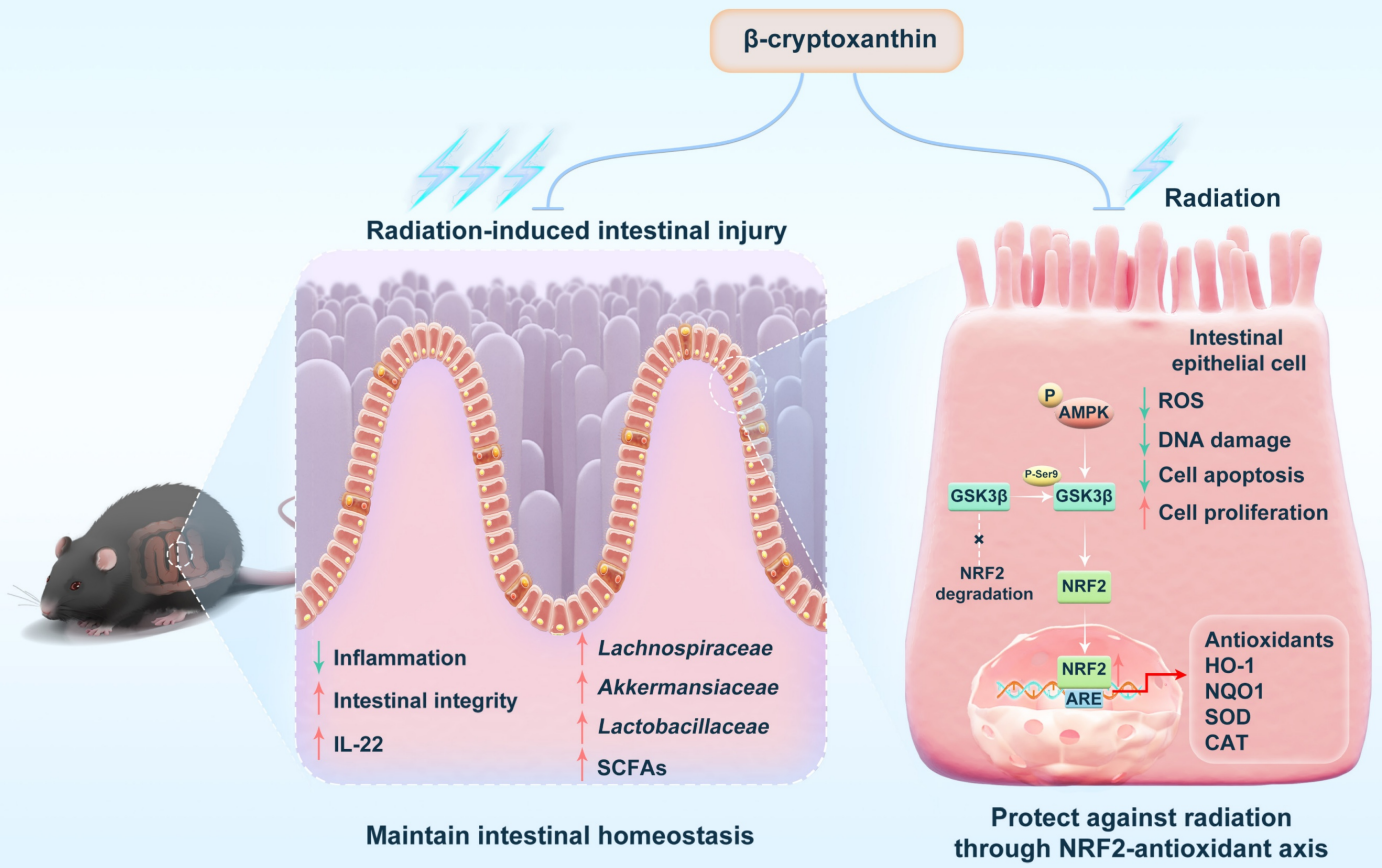
**Schematic illustration of the radioprotective effects and mechanisms of β-cryptoxanthin.** β-Cryptoxanthin ameliorates radiation-induced intestinal injury by maintaining the intestinal homeostasis through enhancing the abundance of beneficial bacteria (such as *Lachnospiraceae*, *Akkermansiaceae*, and *Lactobacillaceae*) and protective products such as SCFAs. On the cell level, β-cryptoxanthin can protect cells against hydrogen peroxide and radiation-induced damage by reducing ROS, promoting proliferation, alleviating apoptosis, and reducing DNA damage. β-Cryptoxanthin can promote the translocation of NRF2 into the nucleus and thus promote the transcription and protein expression of NRF2 target genes, which is regulated by the AMPK-GSK3β signaling pathway.

In this study, we demonstrate that β-cryptoxanthin confers robust radioprotection in both irradiated mice and cells. *In vivo*, β-cryptoxanthin mitigates RIII in mice by promoting crypt cell proliferation and regeneration, activating NRF2-dependent cytoprotective pathways, and increasing IL-22 production. In MODE-K cells, β-cryptoxanthin eliminates radiation-induced ROS, promotes cell proliferation, and reduces apoptosis and DNA damage. Mechanistically, it activates the AMPK-GSK3β axis to drive NRF2 nuclear translocation, thereby enhancing transcription and expression of NRF2 target genes. Additionally, it reprograms gut microbiota to enrich beneficial taxa (e.g., *Lachnospiraceae*, *Akkermansiaceae*, and *Lactobacillaceae*) and elevates protective metabolites such as SCFAs, collectively restoring radiation-disrupted intestinal homeostasis and conferring systemic radioprotection (Fig. 10).

Carotenoids function as biological antioxidants that scavenge ROS, demonstrating potential to prevent or ameliorate diseases including cancer, cardiovascular diseases, and ocular disorders 45-47. Following absorption and metabolism in the small intestine, they are secreted to the lymphatic system, reducing inflammatory responses and exerting multiple protective effects 48, 49. In this study, β-cryptoxanthin enhanced secretion of the anti-inflammatory cytokine IL-22, promoted small intestinal crypt proliferation, reduced epithelial apoptosis, and mitigated RIII—a prevalent complication in abdominal/pelvic radiotherapy 2 that induces gut microbiota dysbiosis and exacerbates intestinal injury 50, 51. In terms of microbiota composition, pretreatment with β-cryptoxanthin increased the abundance of* Lachnospiraceae*, *Akkermansiaceae*, and *Lactobacillaceae* in irradiated mice. *Lachnospiraceae* plays an impotant role in the production of SCFAs and protects against intestinal injury 52. *Akkermansiaceae* is a mucin-degrading probiotic that can produce SCFAs 53, and *Lactobacillaceae* can also produce SCFAs 54, 55. Consistently, β-cryptoxanthin elevated SCFA levels and restored intestinal metabolite homeostasis by remodeling metabolic profiles. These coordinated actions reestablish gut microenvironmental equilibrium, positioning β-cryptoxanthin as a multifunctional modulator of intestinal homeostasis against RIII.

The gut microbiota-modulating effect of β-cryptoxanthin observed in our study is consistent with a broader capacity of carotenoids to shape the intestinal microbial ecosystem. Evidence from various animal models indicates that different carotenoids can promote similar beneficial shifts. For instance, the carotenoids torularhodin and fucoxanthin have been shown to modulate gut microbiota in mice, increasing the abundance of *Akkermansia*, which contributes to ameliorating metabolic disorders 56, 57. Similarly, β-carotene and lycopene can alleviate intestinal inflammation in weaned piglets by increasing potentially beneficial bacterial groups like *Phascolarctobacterium* and *Parasutterella* and decreasing potentially pathogenic bacterial groups like *Treponema*_2 58, 59. Importantly, this microbial modulation is closely associated with the enhanced production of beneficial metabolites, particularly SCFAs, as demonstrated by* in vitro* fermentation studies with β-carotene, lutein, lycopene, and astaxanthin 60, 61. Therefore, the beneficial modulation of gut microbiota composition and function emerges as a shared characteristic of carotenoids. Our findings extend this paradigm into radiobiology, identifying β-cryptoxanthin as a potent agent that employs this microbial regulation to counteract radiation-induced dysbiosis and intestinal injury.

β-Cryptoxanthin mitigates radiation-induced cellular and intestinal injury via the NRF2-antioxidant pathway. NRF2 and its activated antioxidant response elements (AREs) play important roles in defensing oxidative stress and protecting cells against radiation-induced DNA damage 29. Certain carotenoids such as lycopene and astaxanthin promote the transcription and expression of antioxidant genes downstream of NRF2 62, 63. Upon activation, NRF2 translocates into the nucleus and bind to AREs to trigger the expression of ARE-dependent cytoprotective genes 64. Ke et al. demonstrated that β-cryptoxanthin suppresses high glucose-induced oxidative stress in podocyte via activation of the NRF2/HO-1 signaling pathway 65. Zhang et al. found that it maintains mitochondrial function by promoting NRF2 nuclear translocation to inhibit oxidative stress-induced senescence in HK-2 human renal tubular epithelial cells 66. In this study, we demonstrate that β-cryptoxanthin activates NRF2 signaling pathway by promoting NRF2 nuclear translocation following hydrogen peroxide or radiation exposure. Under basal conditions, NRF2 degradation is regulated by two distinct mechanisms: the Keap1/Cul3/Rbx1 complex and additional negative regulators of NRF2, such as Fyn and Bach1. Keap1 acts as a natural inhibitor of NRF2 by binding to NRF2 in the cytosol and promoting ubiquitination and proteasomal degradation of NRF2 67. Notably, β-cryptoxanthin treatment did not alter Keap1 protein levels, indicating NRF2 activation occurs primarily through Keap1-independent mechanisms. In the alternative pathway, GSK3β phosphorylates the NRF2 negative regulator Fyn, leading to Fyn nuclear localization. Fyn subsequently phosphorylates NRF2 at Tyr568, resulting in nuclear export of NRF2 followed by ubiquitination and degradation of NRF2. GSK3β activity suppresses NRF2 nuclear accumulation, while phosphorylation at Ser9 inactivates GSK3β, thereby enhancing NRF2 nuclear translocation 33. GSK3β can be regulated by a number of upstream regulatory factors, including AMPK. AMPK can respond to oxidative stress and ROS 68 and plays important roles in preventing aging and inflammation 69. AMPK may regulate the nuclear translocation of NRF2 through AMPK/GSK3β/NRF2 signaling pathway 33, 34, 67. In MODE-K cells, β-cryptoxanthin pretreatment elevated expression levels of p-AMPK and p-GSK3β (Ser9), indicating AMPK activation and GSK3β inhibition. The results suggest that the nuclear translocation of NRF2 induced by β-cryptoxanthin is regulated by the AMPK-GSK3β signaling pathway. It should be noted that the nuclear translocation of NRF2 promoted by β-cryptoxanthin may also be regulated by other elements that need to be further clarified.

β-Cryptoxanthin, a provitamin A carotenoid present in numerous fruits and vegetables, exhibits multifaceted health benefits including antioxidant defense and cellular signaling modulation 17. As a potent antioxidant, it protects against ROS-induced macromolecular damage and functions as a UV protectant 21. Crucially, β-cryptoxanthin serves as a precursor to vitamin A—an essential nutrient required for vision, growth, development, and immune responses 70. Human epidemiological studies demonstrated that β-cryptoxanthin plays a preventive role against various cancers, with higher serum levels associated with reduced risks of lung 71, gastric 72, and bladder cancers 73. Animal studies confirm its chemopreventive effects against lung 74, gastric 75, and colon cancers 76. The cancer-preventive effects of β-cryptoxanthin may depend on the enhancement of DNA repair as well as antioxidant protection against damage 77, 78. Notably, β-cryptoxanthin demonstrates dual redox properties. Under physiological conditions, it acts as a potent antioxidant regulating cellular oxidative stress. However, in cancer cells with intrinsically elevated intracellular ROS, it may exert pro-oxidant effects triggering ROS-mediated apoptosis 19. The dual radioprotective and anticancer efficacy of β-cryptoxanthin, combined with its superior bioavailability among dietary carotenoids 79, positions it as an ideal radioprotective adjuvant. β-cryptoxanthin-rich foods thus offer practical protection during abdominal/pelvic radiotherapy.

Our results offer a new avenue for the prevention and treatment of RIII, providing a solid theoretical foundation for the clinical translation of β-cryptoxanthin in radioprotection. Future work should include systematic screening of carotenoids with enhanced radioprotective efficacy, with their effects evaluated in both sexes to ensure broad applicability. This approach will accelerate the development of novel therapeutics and expand their potential applications in managing RIII.

## Conclusion

In conclusion, this study identifies β-cryptoxanthin as a novel dual-mechanism radioprotectant that simultaneously activates the AMPK-GSK3β-NRF2 antioxidant axis and reprograms radiation-damaged gut microbiota. Through AMPK-GSK3β-mediated NRF2 nuclear translocation, β-cryptoxanthin alleviates oxidative DNA damage and promotes cell survival in MODE-K mouse intestinal epithelial cells, thereby protecting them from irradiation-induced injury. Orally administered β-cryptoxanthin restores radiation-disrupted gut microbiota and metabolic homeostasis by selectively enriching beneficial bacterial taxa and enhancing production of protective metabolites such as SCFAs, ultimately mitigating RIII. This radioprotective effect is NRF2-dependent, as demonstrated by the complete loss of protection in NRF2 knockout models. Collectively, these findings support the clinical translation of β-cryptoxanthin as an orally active adjuvant to abdominal and pelvic radiotherapy.

## Supplementary Material

Supplementary figures and table.

## Data Availability

The data supporting our findings will be made available from the corresponding author on reasonable request.
