# Supplementary material for: β-Cryptoxanthin Confers Radioprotection against Intestinal Injury via NRF2-Mediated Antioxidant Response and Gut Microbiota Reprogramming
Source: Int J Biol Sci. 2026 Feb 11;22(5):2581–602. doi: 10.7150/ijbs.125307 (PMC12965244; doi:10.7150/ijbs.125307)
Supplement: Supplementary file 1 — Supplementary figures and table. [file ijbsv22p2581s1.pdf]

Supplementary material

β-Cryptoxanthin Confers Radioprotection against Intestinal Injury via NRF2-Mediated Antioxidant Response and Gut Microbiota Reprogramming

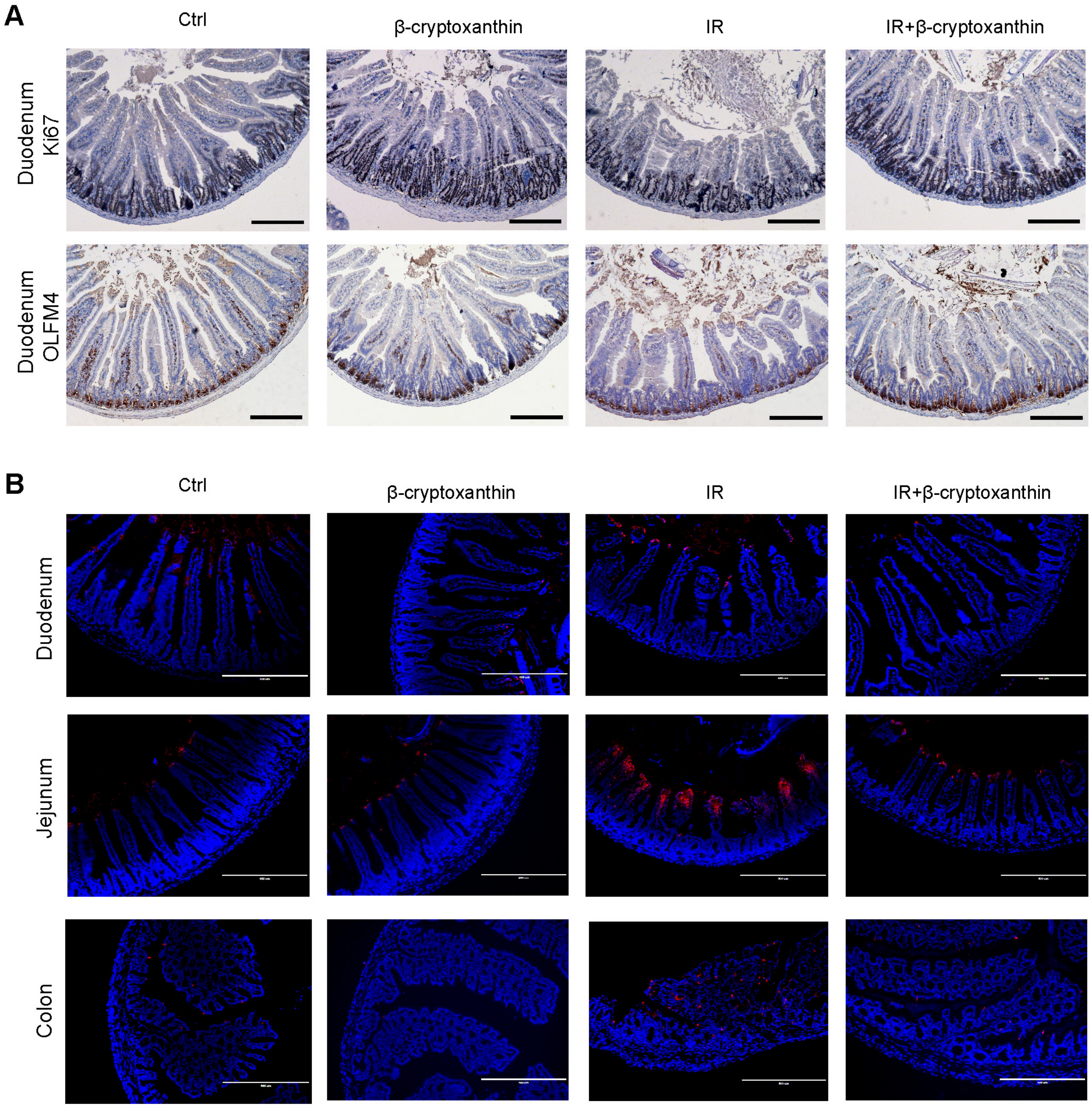

Figure S1. β-Cryptoxanthin promoted proliferation and regeneration of intestinal cells, and reduced apoptosis following ABI. (A) Representative immunohistochemistry images for Ki67 and OLFM4 staining in mouse duodenum on day 3.5 after irradiation. Nucleus were stained with haematoxylin. Scale bars, 100 μm. (B) The effect of β-cryptoxanthin on apoptosis in mouse duodenum, jejunum, and colon tissues was evaluated by TUNEL assays. Nuclei were detected with DAPI. Scale bars, 400 μm. Experiment was repeated three times independently.

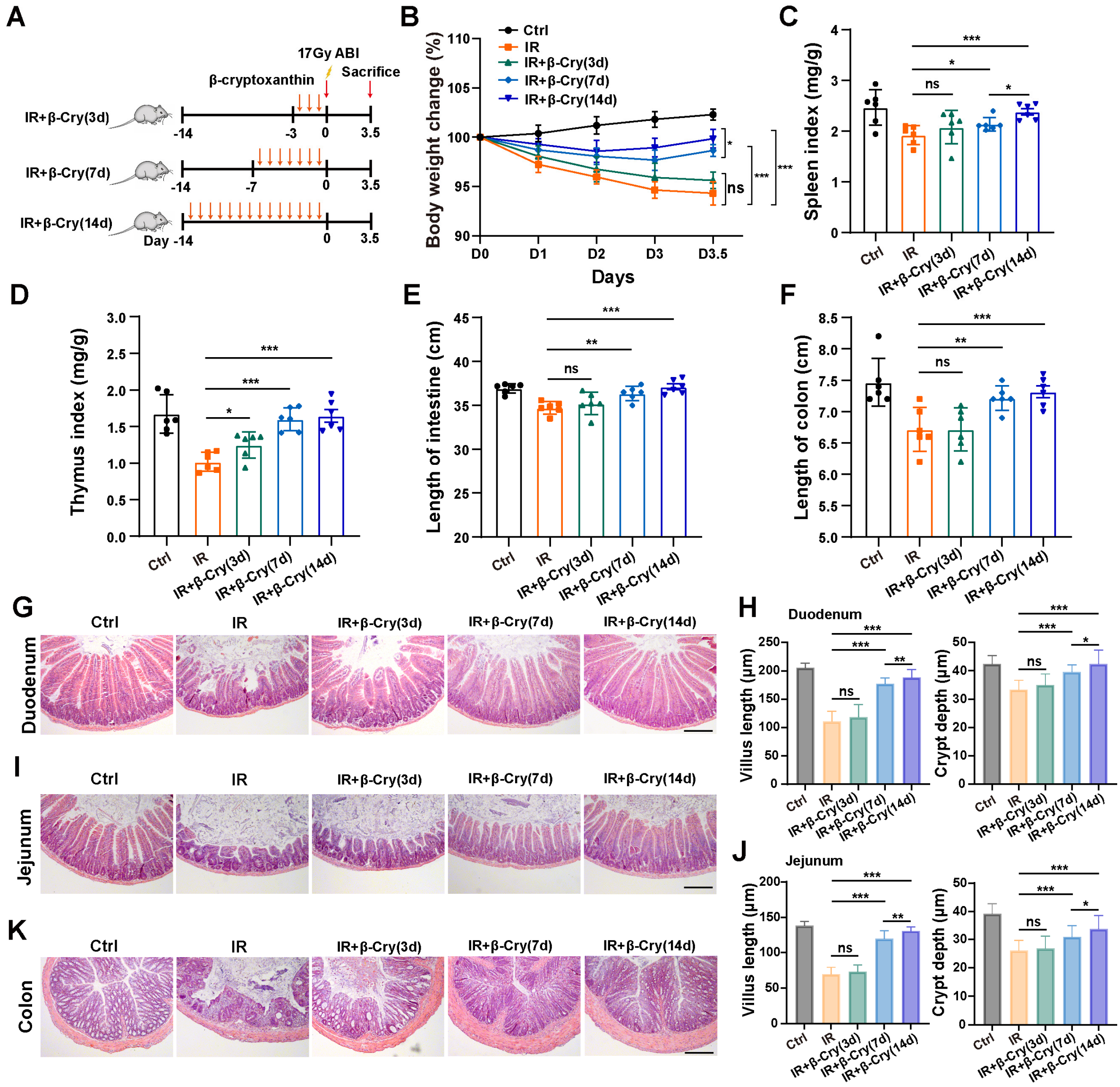

Figure S2. Optimization of β-cryptoxanthin pretreatment duration for radioprotection. (A) Overview of the experimental scheme. Mice (6 per group) were treated with corn oil (Ctrl) or 1 mg/kg/day β-cryptoxanthin for 3, 7, or 14 days and then exposed to 17 Gy ABI. Mice were sacrificed on the 3.5th day. (B) Body weights of mice in different treatment groups over time. (C) Spleen index in different groups. (D) Thymus index in different groups. (E) Length of the small intestines. (F) Length of colons. (G) H&E staining of duodenum sections from mice. (H) Villus length and crypt depth were measured in duodenums. (I) H&E staining of jejunum sections from mice. (J) Villus length and crypt depth were measured in jejunums. (K) H&E staining of colon sections from mice. The scale bar represents 100 μm. β-Cry, β-cryptoxanthin.

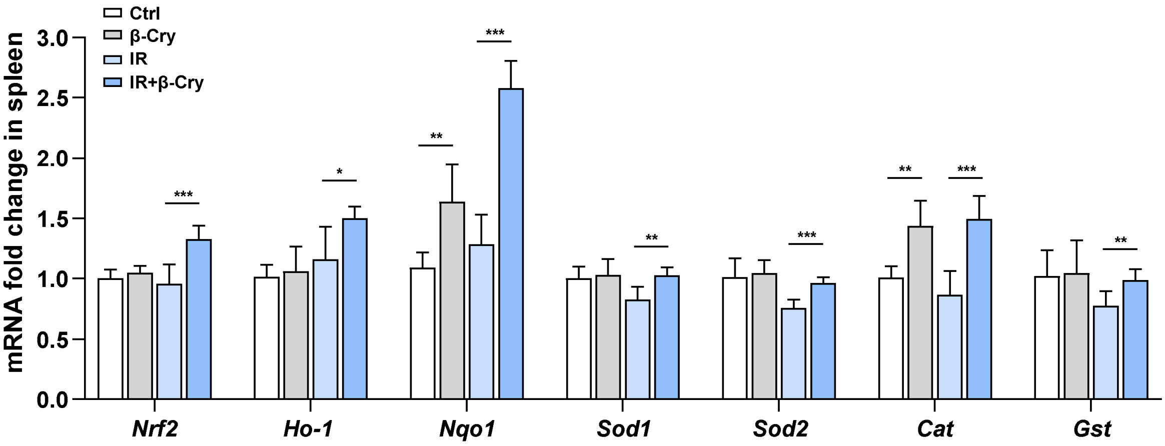

Figure S3. The qPCR analysis of *Nrf2* and its downstream target genes expression in mouse spleen. Data were presented as means ± SD (*n* = 6 per group). β-Cry, β-cryptoxanthin.

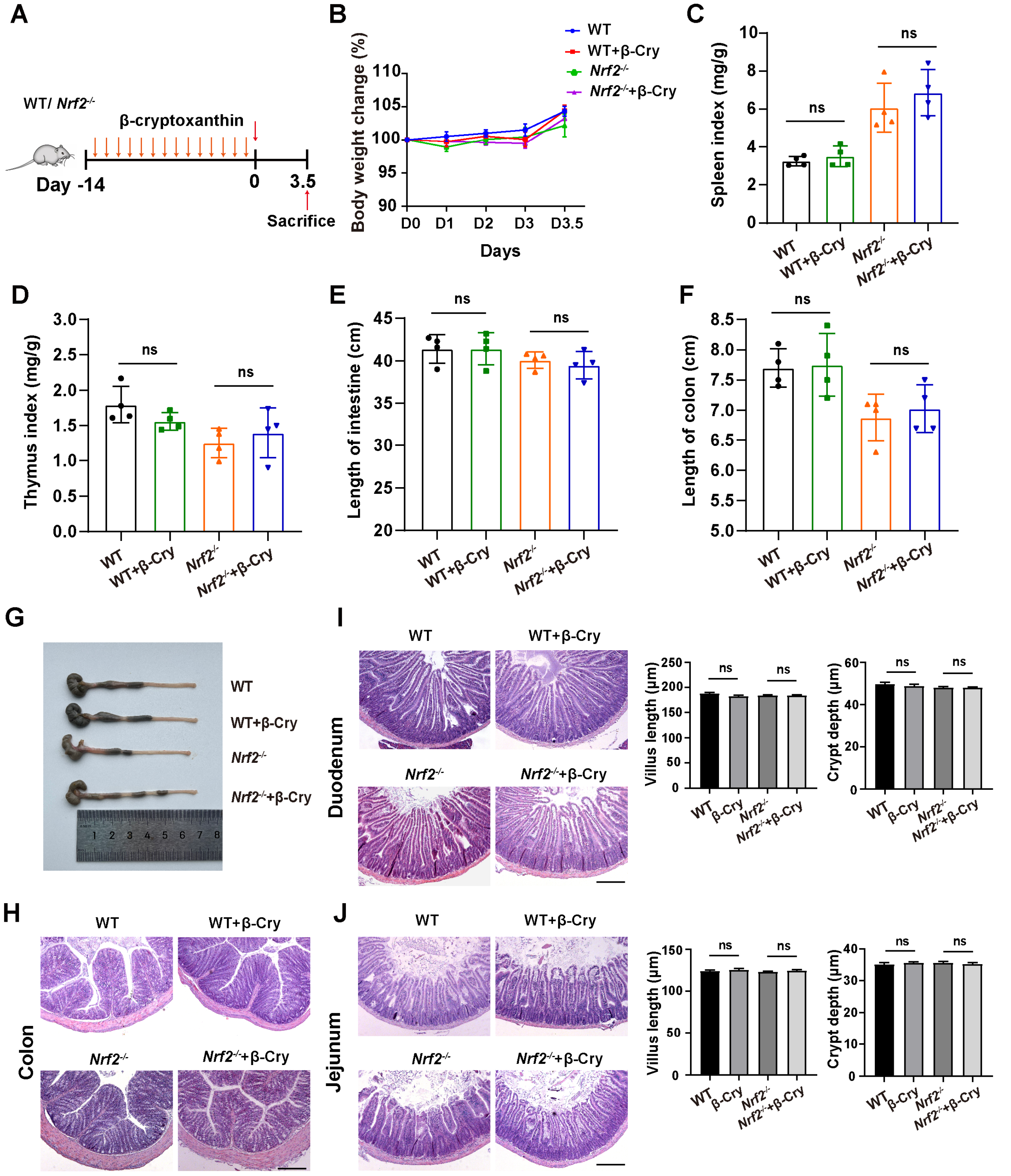

Figure S4. β-Cryptoxanthin had no effect on the intestinal histopathology in either unirradiated WT mice or *Nrf2*$^{-/-}$ mice. (A) Overview of the experimental scheme. Mice (4 per group) were treated with corn oil (Ctrl) or 1 mg/kg/day β-cryptoxanthin for 14 days and then exposed to pseudo-irradiation. Mice were sacrificed on the 3.5th day. Data are means ± SD. (n = 4). (B) Body weights of mice in different treatment groups over time. (C) Spleen index in different groups. (D) Thymus index in different groups. (E) Length of the small intestines. (F-G) Length of colons and representative image of colons from mice across the

different treatment groups on day 3.5 after 17 Gy ABI. (H) HE staining of colon sections from mice. The scale bar represents 100 μm. (I-J) HE staining of duodenum and jejunum sections from mice. The scale bar represents 100 μm. Intestinal villus length and crypt depth were measured and shown at the right panel. β-Cry, β-cryptoxanthin.

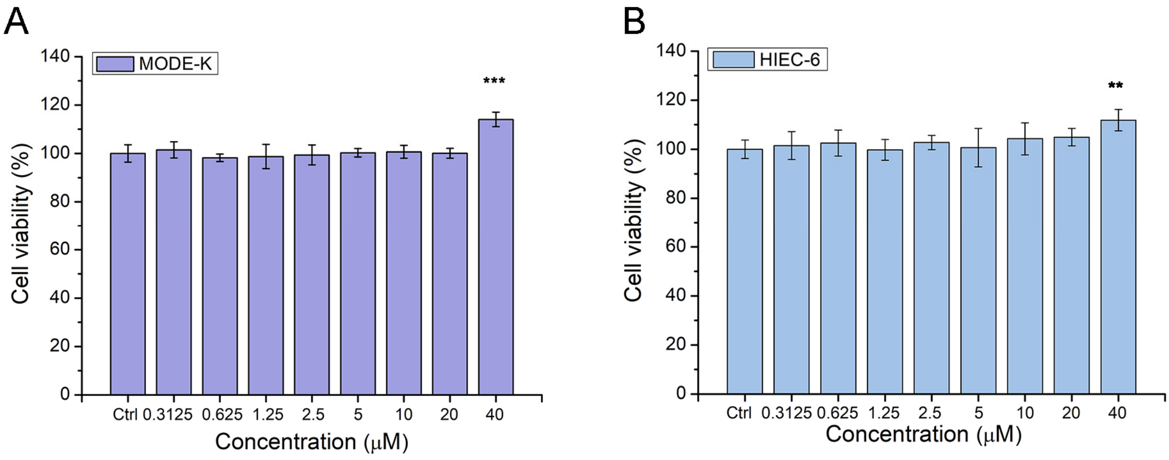

Figure S5. Cytotoxicity of β-cryptoxanthin to MODE-K (A) and HIEC-6 (B) cells for 48 h. Cell viability was measured using the CCK-8 assay. The results are the mean ± SD of three experiments, each in triplicate.

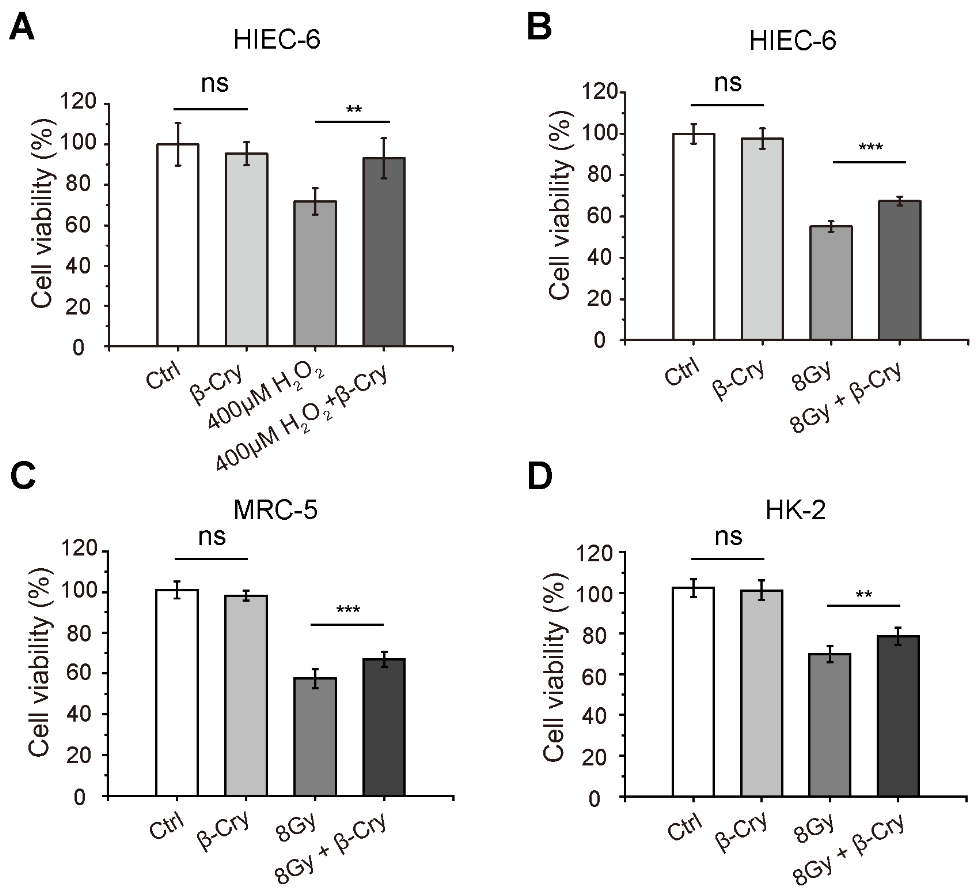

Figure S6. β-Cryptoxanthin has radioprotective effects on HIEC-6, MRC-5, and HK-2 cells. (A) Effect of β-cryptoxanthin pretreatment on cell survival after hydrogen peroxide treatment of HIEC-6 cells. (B-D) Effect of β-Cryptoxanthin pretreatment on cell survival after radiation treatment of HIEC-6 (B), MRC-5(C), and HK-2 (D) cells. The results are the mean ± SD of three experiments, each in triplicate.

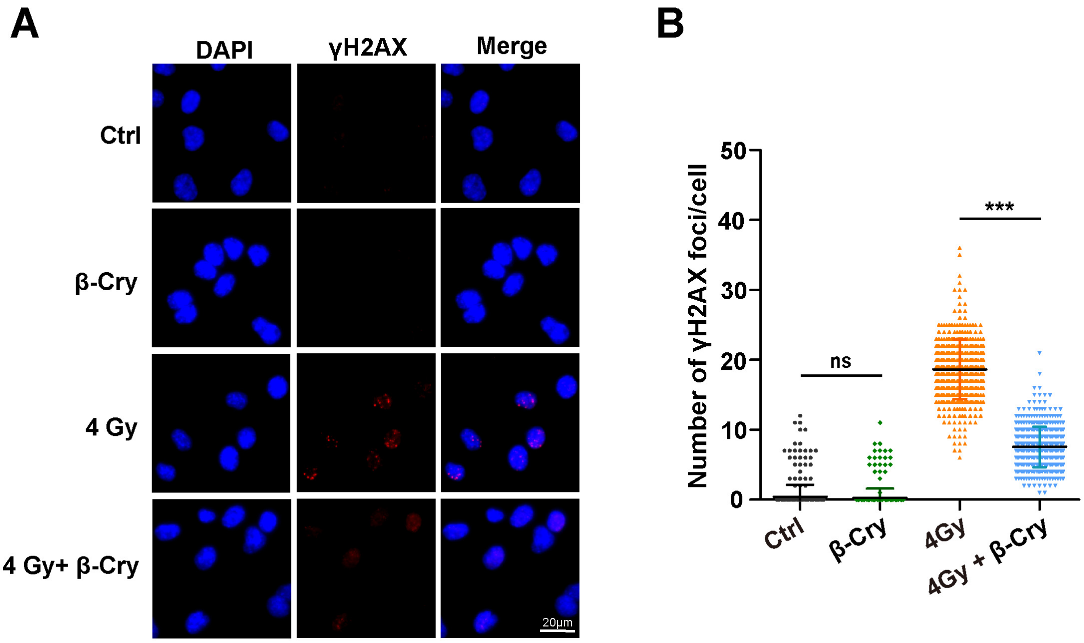

Figure S7. Representative images and quantitative analysis of immunofluorescence staining for γH2AX foci at 12 h post-irradiation. (A) Representative images of immunofluorescence staining for γH2AX foci (red dots). MODE-K cells were pretreated with β-cryptoxanthin for 12 h and then exposed to 4 Gy IR. γH2AX foci were counted at 12 h post-irradiation. Scale bars, 20 μm. (B) Quantitative analysis of γH2AX foci (red) numbers per cell. Data are means ± SD, with more than 150 cells were counted from three independent experiments. β-Cry, β-cryptoxanthin.

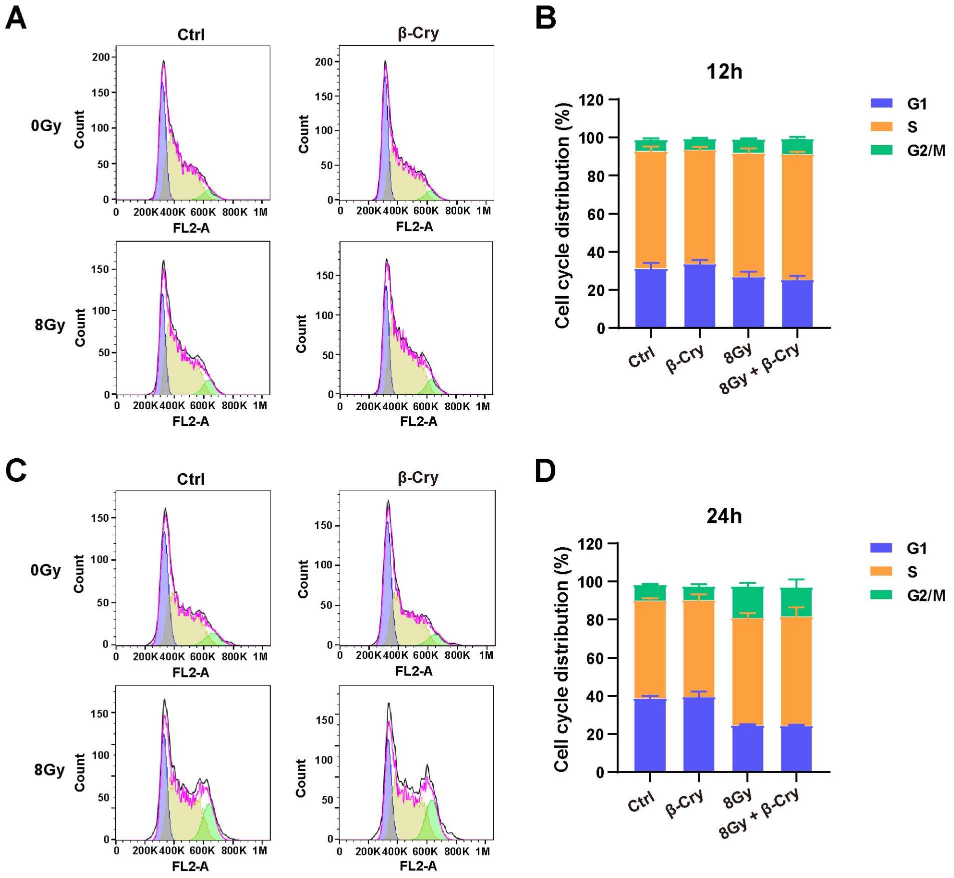

Figure S8. **Effect of β-cryptoxanthin pretreatment on cell cycle distribution in MODE-K cells.** (A-B) Flow cytometry histograms and statistic analysis of the cell cycle in MODE-K cells at 12 h post-irradiation. (C-D) Flow cytometry histograms and statistic analysis of the cell cycle in MODE-K cells at 24 h post-irradiation. Data are shown as the mean ± SD of three independent experiments.

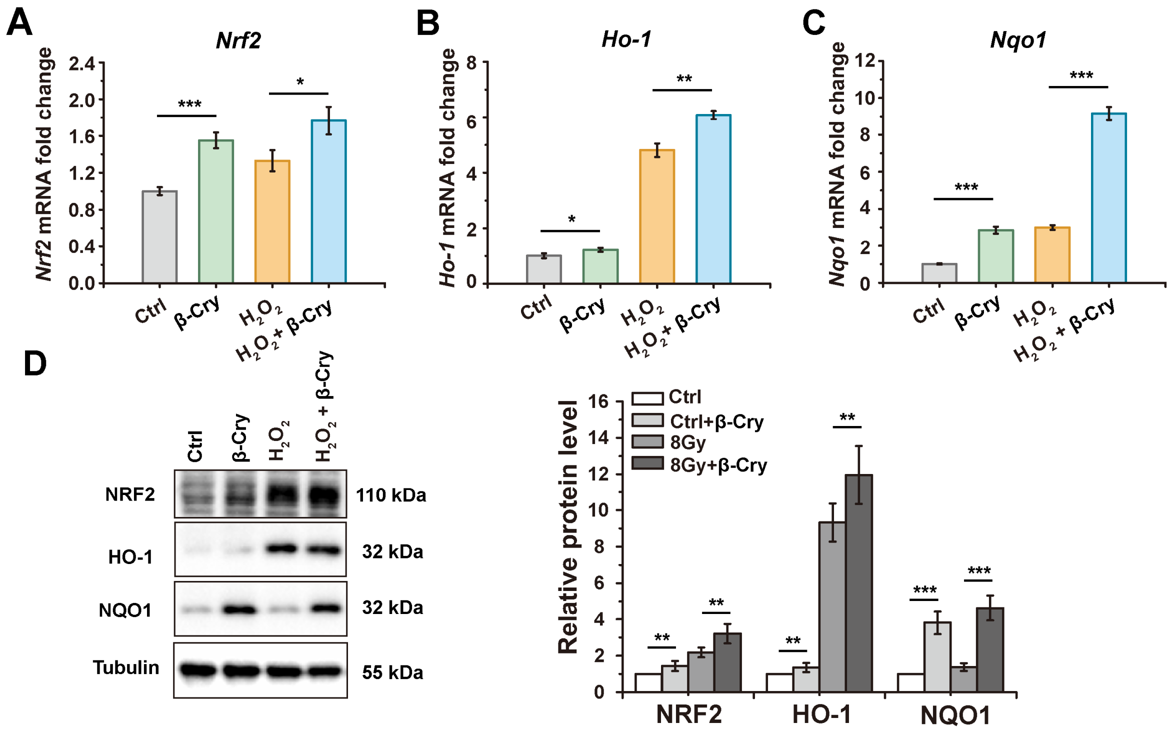

Figure S9. Pretreatment with β-cryptoxanthin before exposure to hydrogen peroxide enhanced the NRF2-mediated antioxidant pathway in MODE-K cells. (A-C) The mRNA levels of *Nrf2*, *Ho-1*, and *Nqo1* in MODE-K cells were determined by qPCR. Cells were pretreated with β-cryptoxanthin before exposure to 400 μM hydrogen peroxide and harvested at 8 h after IR. (D) Western blotting of NRF2, HO-1 and NQO1 in MODE-K cells pretreated with β-cryptoxanthin and then exposed to 400 μM hydrogen peroxide. Tubulin was a loading control. Quantitative analysis of expression of the proteins was conducted using Image Lab software (version 6.1, Bio-rad). The data are representative of three independent experiments. β-Cry, β-cryptoxanthin.

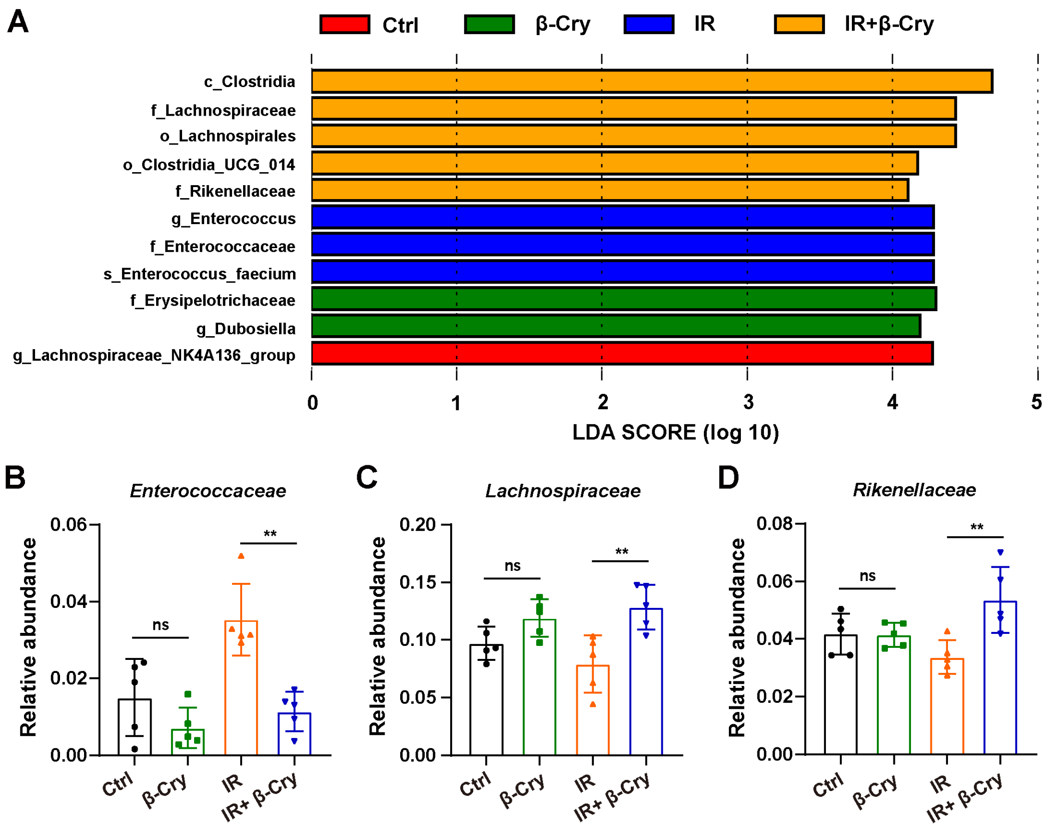

Figure S10. Identification of discriminative microbial biomarkers by LEfSe analysis. (A) Histogram of LDA scores for the most discriminative taxa. (B-D) The relative abundance of *Enterococcaceae* (B), *Lachnospiraceae* (C), and *Rikenellaceae* (D). Data were presented as means ± SD (n = 6 per group). β-Cry, β-cryptoxanthin.

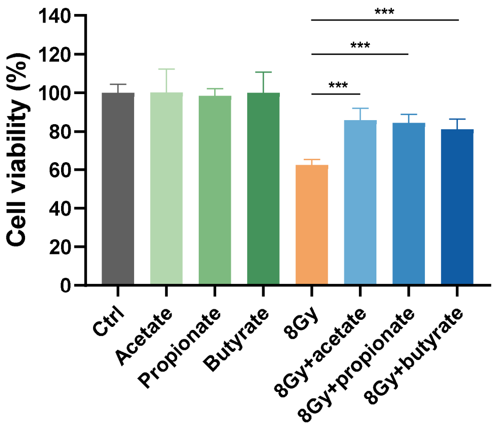

Figure S11. SCFAs protect intestinal epithelial cells from radiation-induced damage. MODE-K cells were pretreated with acetate (5 mM), propionate (1 mM), or butyrate (1 mM) for 24 h and then exposed to 8 Gy γ-irradiation. Cell viability was assessed 24 h post-irradiation using the CCK-8 assay. Data are presented as mean ± SD ($n \geq 3$).

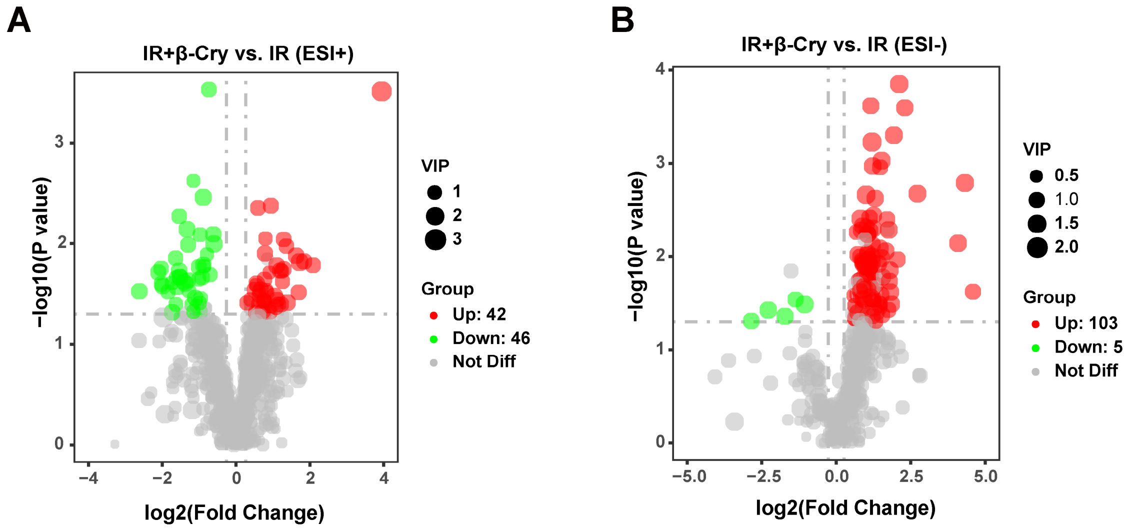

Figure S12. Volcano plots showing differential metabolites in fecal samples upon β-Cryptoxanthin treatment in irradiated mice obtained by LC-MS/MS ES+ (A) and ES- (B). Each dot in the volcano plots represents a detected metabolite. Metabolites that significantly increase and decrease are shown in red and green, respectively.

Table S1. Primer sequences used for quantitative real-time PCR

Gene	Forward primer (5’→3’)	Reverse primer (5’→3’)
Nrf2	TAGATGACCATGAGTCGCTT	GCCAAACTTGCTCCATGTCC
Ho-1	CACTCTGGAGATGACACCT	GTGTTCCTCTGTCAGCATCACC
Nqo1	AGAGAGTGCTCGTAGCAGG	GTGGTGATAGAAAGCAAGGTCTT
Sod1	AACCAGTTGTGTTGTCAGG	CCACCATGTTTCTTAGAGTGAGG
Sod2	CCAAGGGAGATGTTACAAC	GGGCTCAGGTTTGTCCAGAA
Cat	AGCGACCAGATGAAGCAGT	TCCGCTCTCTGTCAAAGTGTG
Gst	TGCGACCGCATTCCAGAGG	TCCACCTTCTCGTCAGTGCGAA
Gapdh	AGGTCGGTGTGAACGGATT	TGTAGACCATGTAGTTGAGGTCA
